# The odontoblastic differentiation of dental mesenchymal stem cells: molecular regulation mechanism and related genetic syndromes

**DOI:** 10.3389/fcell.2023.1174579

**Published:** 2023-09-25

**Authors:** Houwen Pan, Yiling Yang, Hongyuan Xu, Anting Jin, Xiangru Huang, Xin Gao, Siyuan Sun, Yuanqi Liu, Jingyi Liu, Tingwei Lu, Xinyu Wang, Yanfei Zhu, Lingyong Jiang

**Affiliations:** ^1^ Center of Craniofacial Orthodontics, Department of Oral and Cranio-Maxillofacial Surgery, Shanghai Ninth People’s Hospital, Shanghai Jiao Tong University School of Medicine, Shanghai, China; ^2^ College of Stomatology, Shanghai Jiao Tong University, Shanghai, China; ^3^ National Center for Stomatology, Shanghai, China; ^4^ National Clinical Research Center for Oral Disease, Shanghai, China; ^5^ Shanghai Key Laboratory of Stomatology, Shanghai, China; ^6^ Shanghai Research Institute of Stomatology, Shanghai, China

**Keywords:** odontoblastic differentiation, dental mesenchymal stem cells, molecular regulation, signaling pathway, genetic syndrome

## Abstract

Dental mesenchymal stem cells (DMSCs) are multipotent progenitor cells that can differentiate into multiple lineages including odontoblasts, osteoblasts, chondrocytes, neural cells, myocytes, cardiomyocytes, adipocytes, endothelial cells, melanocytes, and hepatocytes. Odontoblastic differentiation of DMSCs is pivotal in dentinogenesis, a delicate and dynamic process regulated at the molecular level by signaling pathways, transcription factors, and posttranscriptional and epigenetic regulation. Mutations or dysregulation of related genes may contribute to genetic diseases with dentin defects caused by impaired odontoblastic differentiation, including tricho-dento-osseous (TDO) syndrome, X-linked hypophosphatemic rickets (XLH), Raine syndrome (RS), hypophosphatasia (HPP), Schimke immuno-osseous dysplasia (SIOD), and Elsahy-Waters syndrome (EWS). Herein, recent progress in the molecular regulation of the odontoblastic differentiation of DMSCs is summarized. In addition, genetic syndromes associated with disorders of odontoblastic differentiation of DMSCs are discussed. An improved understanding of the molecular regulation and related genetic syndromes may help clinicians better understand the etiology and pathogenesis of dentin lesions in systematic diseases and identify novel treatment targets.

## 1 Introduction

Dentin is a thick and highly mineralized tissue layer under the enamel that protects the dental pulp cavity from infections, supports and provides nutrition to the enamel, and alleviates dental pressure (Lopez-Cazaux et al., 2006; Liu et al., 2022b). They are formed from odontoblasts (Zhang et al., 2005; Martens et al., 2013). Dentin formation, also known as dentinogenesis, begins with the differentiation of odontoblasts. Odontoblasts are derived from the neural crest-derived mesenchymal cells (Cobourne and Sharpe, 2003). Odontoblasts first occur at the principal cusp tip and then proceed to the base of the tooth, suggesting a spatiotemporal pattern of odontoblast differentiation (Thesleff, 2003; Chen et al., 2008). Odontoblastic differentiation is regulated by a network encompassing signaling pathways, transcriptional factors (TFs), and posttranscriptional and epigenetic regulation. However, any problems in the regulatory network affect dentin development, most of which appear to be genetic syndromes with dentin defects caused by impaired odontoblast differentiation. Therefore, the molecular regulatory mechanism of odontoblastic differentiation of dental mesenchymal stem cells (DMSCs) is discussed in this review, and genetic syndromes associated with odontoblastic differentiation-related dentin lesions are also discussed.

## 2 DMSCs with odontoblastic differentiation ability

**FIGURE 1:**
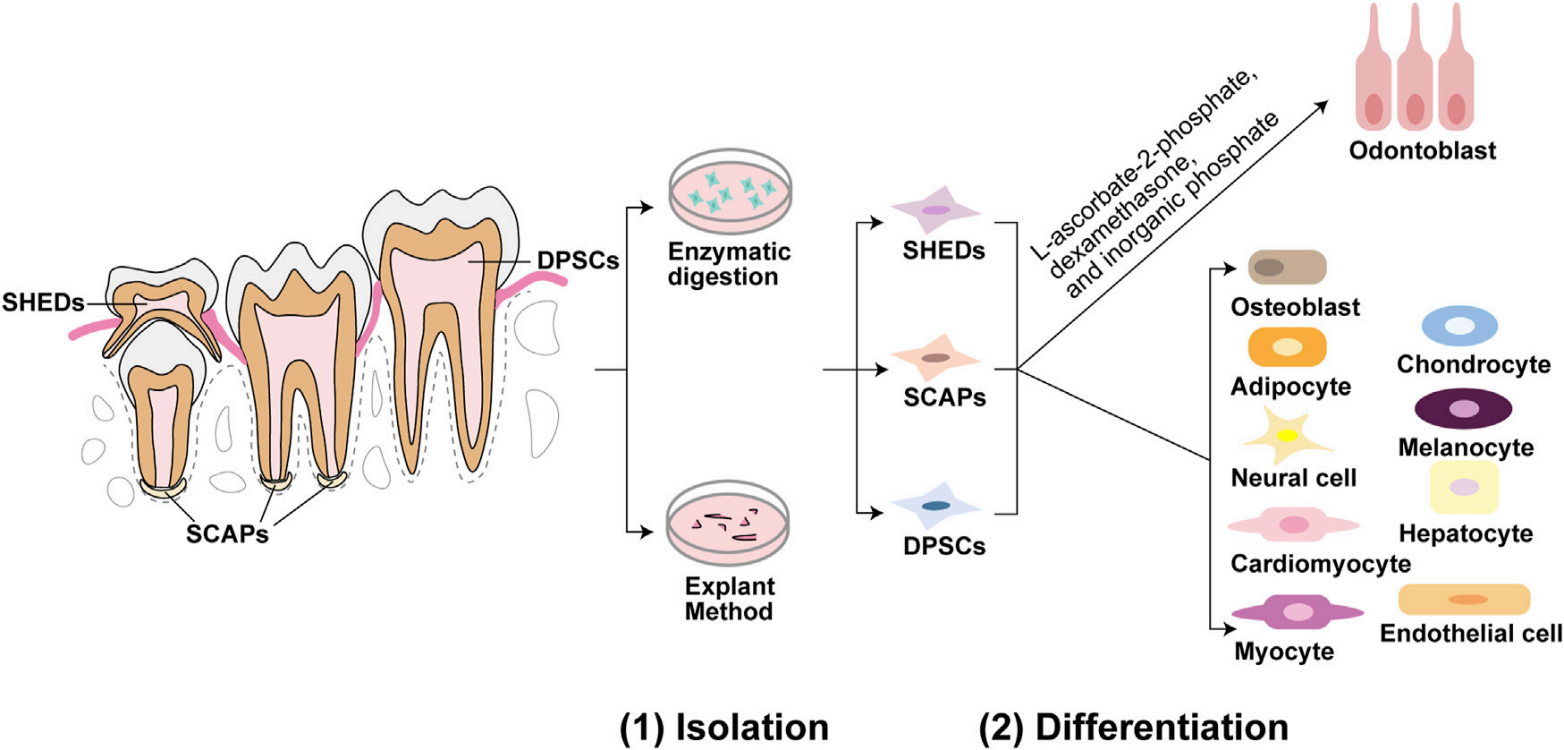
Isolation and differentiation of dental mesenchymal stem cells (DMSCs). Dental pulp stem cells (DPSCs) are located in dental pulp of permanent teeth, stem cells from human exfoliated deciduous teeth (SHEDs) are located in immature dental pulp of deciduous teeth, and stem cells from apical papilla (SCAPs) are located in root apical papilla tissue on the exterior of the root foramen area. These three DMSCs can be isolated by enzymatic solution and explant method. DPSCs, SHEDs, and SCAPs can be cultured in the same induction medium of L-ascorbate-2-phosphate, dexamethasone, and inorganic phosphate to undergo the odontoblastic differentiation process. While under appropriate induction conditions, they can differentiate into a variety of cells besides odontoblasts. For example, DPSCs can differentiate into odontoblasts, osteoblasts, adipocytes, neural cells, cardiomyocytes, myocytes, chondrocytes, melanocytes, and hepatocytes. SHEDs can differentiate into osteogenic, chondrogenic, adipogenic cells, neural cells, odontoblasts, endothelial cells, and hepatocytes. And SCAPs can differentiate into adipocytes, odontoblasts, and osteoblasts.

In the last two decades, various populations of mesenchymal stem cells have been identified in dental tissues, such as dental pulp stem cells (DPSCs), stem cells from human exfoliated deciduous teeth (SHEDs), periodontal ligament stem cells (PDLSCs), dental follicle precursor cells (DFPCs), alveolar bone-derived mesenchymal stem cells (ABMSCs), stem cells in the apical papilla of human immature permanent teeth (SCAPs, stem cells from apical papilla), tooth germ progenitor cells (TGPCs), and gingiva-derived mesenchymal stem cells (GMSCs) (Gronthos et al., 2000; Miura et al., 2003; Seo et al., 2004; Morsczeck et al., 2005; Matsubara et al., 2005; Sonoyama et al., 2006; Ikeda et al., 2008; Zhang et al., 2009). Multipotency is an important feature of DMSCs (Nuti et al., 2016). Different genes and microenvironments that contain specific signals, including biochemical, biomechanical and biophysical factors such as growth factors, signaling pathways and other molecules, can modulate cell fate specifications of DMSCs (Mitsiadis et al., 2011; Rodríguez-Lozano et al., 2011; Marrelli et al., 2018). Under appropriate induction conditions, DMSCs can differentiate into several cell lineages, including odontoblasts, osteoblasts, chondrocytes, neural cells, myocytes, cardiomyocytes, adipocytes, endothelial cells, melanocytes, and hepatocytes (Nuti et al., 2016; Zhang et al., 2006; d’Aquino et al., 2007; Carinci et al., 2008; Armiñán et al., 2009; Paino et al., 2010; Gan et al., 2020), as shown in Figure 1. Odontoblastic differentiation ability is a critical characteristic of DMSCs. Therefore, we mainly discuss DMSCs with odontoblastic differentiation abilities, including DPSCs, SHEDs, and SCAPs. Gronthos et al. first isolated DPSCs from adult human pulp in 2000 and in 2003 Miura et al. identified SHEDs (Gronthos et al., 2000; Miura et al., 2003). Sonoyama et al. discovered a novel type of stem cell in the apical papilla of immature human permanent teeth, known as SCAPs (Sonoyama et al., 2006). DPSCs are a cell population with a high proliferation rate, the ability to renew, and the potential to differentiate into various cell types (Gronthos et al., 2000). DPSCs are derived from the migrating neural crest cells in the ectoderm and have characteristics similar to those mesenchymal stem cells (MSCs) (Nuti et al., 2016). In contrast to the properties of DPSCs, the proliferation rate and osteo-inducing ability *in vivo* of SHEDs is higher. However, a complete dentin-pulp-like complex cannot be reconstituted using SHEDs (Miura et al., 2003). SHEDs possess multiple differentiation capacities, including odontoblastic, osteogenic, neurogenic, adipogenic, myogenic, and chondrogenic, endothelial, and hepatocytic differentiation (Miura et al., 2003; Sakai et al., 2010; Yamaza et al., 2010; Ishkitiev et al., 2012; Martens et al., 2013). The expression of STRO-1, a surface marker in apical papilla cells, was positive in SCAPs. This indicates the presence of SCAPs in dental apical tissues (Huang et al., 2008). Compared to DPSCs, SCAPs possess a higher tissue regeneration ability (Martens et al., 2013). SCAPs exhibit osteogenic/odontogenic, neurogenic, and adipogenic differentiation (Sonoyama et al., 2006; Martens et al., 2013). Moreover, tooth formation ceased in the absence of SCAPs (Huang et al., 2008). Cultured in L-ascorbate-2-phosphate, dexamethasone, and inorganic phosphate, DMSCs can differentiate into odontoblasts *in vitro* (Gronthos et al., 2000; Miura et al., 2003; Sonoyama et al., 2006) (Figure 1). DMSCs are thought to be an accessible stem cell source, as there is no risk for the donor and they are non-controversial (Ko et al., 2020), and DMSCs may be a potential source for dentin regeneration.

## 3 Molecular mechanisms regulating odontoblastic differentiation

### 3.1 Signaling pathways

#### 3.1.1 Wnt signaling pathway

The first member of the Wnt family, the *Wnt1* gene, was discovered by Nusse and Varmus in 1982 (Nusse and Clevers, 2017), since then, there have been numerous studies on the Wnt pathway. The Wnt signal transduction cascade is critical for the regulation of development, control of stem cells, and disease (Nusse and Clevers, 2017). Moreover, the Wnt pathway is important for odontoblast differentiation (Chen et al., 2009a).

Wnt/β-catenin signaling pathway is composed of receptors, activators, inhibitors, modulators, kinases, and phosphatases (Duan and Bonewald, 2016; Tamura and Nemoto, 2016). Wnt proteins are classified into two groups: one group is canonical Wnts and the other is non-canonical Wnts. Canonical Wnts, including Wnt1, Wnt2, Wnt3, Wnt3a, and Wnt7a are β-catenin-dependent, whereas non-canonical Wnts are independent of β-catenin or inhibit the canonical β-catenin pathway, including Wnt4, Wnt5a, Wnt5b, Wnt6, and Wnt11 (Tamura and Nemoto, 2016). In the cytomembrane, Wnt proteins bind to frizzled receptor (FZD) and LDL receptor-related protein 5/6 (LRP5/6), which is a receptor complex of two signal-transducing molecules, as shown in Figure 4 (Janda et al., 2017; Nusse and Clevers, 2017). When Wnt signaling is activated, β-catenin is stabilized and accumulates in the nucleus, where it interacts with T cell-specific factor/lymphoid enhancer-binding factor 1 (TCF/Lef1) and transcriptional coactivators regulating its target genes (Clevers and Nusse, 2012; Baron and Kneissel, 2013; Steinhart and Angers, 2018). While Wnt/β-catenin pathway is regulated by its antagonists, including proteins such as Notum, Dickkopf (DKK) and the Sclerostin/SOST families (Cruciat and Niehrs, 2013; Kakugawa et al., 2015), and Wnt target genes such as *Rnf43* and *Znrf3* (Hao et al., 2012; Koo et al., 2012). The expression of pathway components demonstrated the involvement of Wnt/β-catenin signaling in odontoblastic differentiation. Wnt3, Wnt4, Wnt6, Wnt7b, and Wnt10b are expressed in the epithelium, whereas Wnt5a is locally expressed in the dental papillae and mesenchyme (Wang et al., 2014a).

The canonical Wnt/β-catenin pathway plays an important role in odontoblastic differentiation. Wnt10a mediates expression of dentin sialophosphoprotein (*Dspp*), an upstream regulatory molecule. It is critical for dentinogenesis and odontoblastic differentiation (Yamashiro et al., 2007). Lef1 is important for odontoblast differentiation because it upregulates *DSPP* and osteocalcin (*OCN*) mRNA expression in dental pulp cells (DPCs) (Yokose and Naka, 2010). β-catenin knockdown results in decreased odontoblastic differentiation. Mechanistically, β-catenin activates runt-related transcription factor 2 (Runx2), thereby enhancing odontoblastic differentiation of DPCs during reparative dentin formation (Han et al., 2014). *In vitro* study has suggested that β-catenin signaling enhances the formation of pre-odontoblasts. The number of pre-odontoblasts and odontoblasts increased after the exposure of DPCs to Wnt3a. Expression of dentine matrix protein 1 (*Dmp1*) and *Dspp* is upregulated in DPCs exposed to Wnt3a (Vijaykumar et al., 2021). *In vivo* stem cell implantation assay suggested that the synergistic action of bone morphogenetic protein 9 (BMP9) and Wnt3a may enhance the odontoblastic differentiation of immortalized mouse stem cells of the apical papilla tissue of mouse lower incisor teeth (iSCAPs) (Zhang et al., 2015a). Wntless (Wls) is a Wnt chaperone protein that is essential for Wnt signaling. Deletion of the *Wls* gene reduces activation of the Wnt pathway and downregulates Runx2 levels, thereby disrupting the homeostasis of odontoblast differentiation (Lim et al., 2014). Odontoblast-specific deletion of the *Wls* gene leads to the downregulation of Wnt10a, β-catenin, collagen type I (Col1), and dentin sialoprotein (DSP), leading to reduced canonical Wnt activity and inhibition of odontoblast maturation (Bae et al., 2015). *In vivo* study showed that the deletion of *Wls* or overexpression of the Wnt antagonist *Dkk1* decreased odontoblastic differentiation by inhibiting Wnt signaling (Zhang et al., 2021b). These results demonstrate the involvement of β-catenin pathway in odontoblastic differentiation.

**FIGURE 2:**
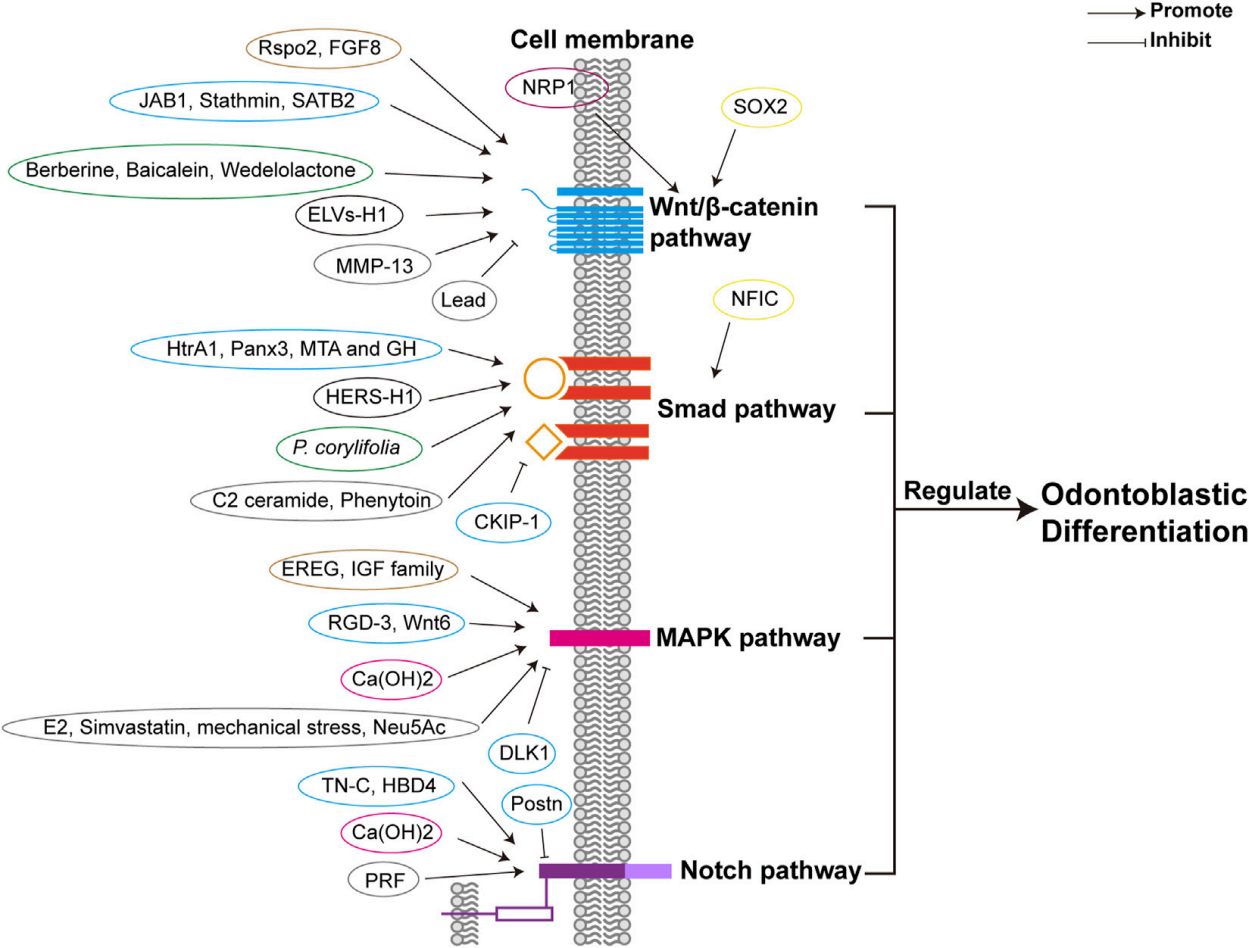
Different factors regulate odontoblastic differentiation via signaling pathways. Different factors are classified in different colors according to their properties, including transcriptional factors (yellow), growth factors (brown), proteins (blue), receptors (purple), herbal extractions (green), other cell lineages (black), calcium compound (rose), and others (such as proteinase, exosome-like vesicles, lipid, mechanical stress, other organic compounds, etc.) (grey). The four signaling pathways are depicted respectively in blue, orange, pink, and purple. The receptors of the signaling pathways are the same as those in Figure 4. Abbreviations: Smad: Small mothers against decapentaplegia; MAPK: Mitogen-activated protein kinase; Notch: Neurogenic locus notch homolog; SOX2: SRY-box 2; Rspo2: R-spondin 2; FGF8: Fibroblast growth factor 8; JAB1: Jun activation domain-binding protein 1; NRP1: Neuropilin-1; MMP-13: Matrix-metalloproteinase-13; ELVs-H1: Exosome-like vesicles derived from a Hertwig’s epithelial root sheath (HERS) cell line; SATB2: Special AT-rich sequence-binding protein 2; NFIC: nuclear factor I-C; HtrA1: High-temperature requirement protein A1; MTA: Mineral trioxide aggregate; GH: Growth hormone; Panx3: Pannexin3; *P. corylifolia: Psoralea corylifolia L.*; CKIP-1: Casein kinase 2 interacting protein-1; EREG: Epiregulin; E2: Estrogen 17β-estradiol; RGD-3: Arginine-glycine-aspertic acid (RGD) peptide-3; IGF: Insulin-like growth factor; Ca(OH)_2_: Calcium hydroxide; Neu5Ac: N-acetylneuraminic acid; DLK1: Delta-like homologue 1; TN-C: Tenascin-C; HBD4: Human β-defensin 4; PRF: Platelet-rich fibrin; Postn: Periostin.

Different factors, including TFs, growth factors, proteins, herbal extracts, and exosome-like vesicles can promote odontoblastic differentiation by enhancing the canonical Wnt/β-catenin pathway, as shown in Figure 2 and Table 1. Increased expression of SRY-box 2 (SOX2) promotes odontoblastic differentiation of DPSCs via the Wnt pathway, in which *Wnt* genes are upregulated in DPSCs (Yang et al., 2017). R-spondin 2 (Rspo2), a stem cell growth factor, promotes the proliferation and odontogenic differentiation of hDPSCs via Wnt/β-catenin pathway (Gong et al., 2020). Fibroblast growth factor 8 (FGF8), an effective stimulator of canonical Wnt/β-catenin signaling, is also essential for tooth development. Upregulated expression of odontoblast proteins, Runx2, Osterix (Osx), and Ocn were observed in primary cultured mesenchymal cells treated with the CHIR99021 (glycogen synthase kinase 3β (GSK3β) inhibitor) and FGF8 (Kimura et al., 2022). Odontoblastic differentiation of DPSCs can be promoted by increased expression of Jun activation domain-binding protein 1 (JAB1), a multipotent protein stabilizing proteins and controlling cell proliferation, via Wnt/β-catenin signaling, as the expression of GSK3β and β-catenin was dramatically increased (Lian et al., 2016). Neuropilin-1 (NRP1) induces odontoblast differentiation of DPSCs via the classical Wnt/β-catenin pathway, which upregulates nuclear β-catenin expression (Song et al., 2017). Stathmin can enhance the odontogenetic differentiation of hDPSCs via Wnt/β-catenin pathway (Zhang et al., 2019). Matrix-metalloproteinase-13 (MMP-13) interacts with Wnt/β-catenin pathway, as in *MMP13*-knockout mice, the Wnt-responsive gene *Axin2* was downregulated and dentin formation was defected (Duncan et al., 2022). Herbal extracts also promote odontoblast differentiation of DPSCs via Wnt/β-catenin pathway, such as Berberine, Baicalein, and Wedelolactone (Lee et al., 2016; Wang et al., 2018; Wu et al., 2019). Exosome-like vesicles derived from the Hertwig’s epithelial root sheath (HERS) cell line (ELVs-H1) boosts the migration and proliferation of DPCs. And ELVs-H1 also promotes odontogenic differentiation through activation of the Wnt/β-catenin pathway (Zhang et al., 2020b). Suppression of Wnt/β-catenin pathway is related to the inhibition of odontoblastic differentiation. Knockdown of special AT-rich sequence-binding protein 2 (SATB2) leads to decreased β-catenin levels and increased DKK1 expression, resulting in the inhibition of odontoblastic differentiation of hDPSCs (Xin et al., 2021). Lead (Pb) inhibits Wnt/β-catenin pathway and thus impairs odontoblastic differentiation of hDPSC (Khalid et al., 2022).

**TABLE 1:** Factors regulating odontoblastic differentiation via signaling pathways.

Signaling pathways	Factors	Definitions	Effects	References
Wnt pathway	SOX2	SRY-box 2, an important transcription factor with important significance in maintaining pluripotency of stem cells and somatic cell reprogramming	Promote	Yang et al. (2017)
	Rspo2	R-spondin 2, a potent stem cell growth factor which strongly potentiate Wnt/β-catenin signaling	Promote	Gong et al. (2020)
	FGF8	Fibroblast growth factor 8	Promote	Kimura et al. (2022)
	JAB1	Jun activation domain-binding protein 1, a multipotent protein stabilizing proteins and controlling cell proliferation	Promote	Lian et al. (2016)
	NRP1	Neuropilin-1, a single-pass transmembrane glycoprotein	Promote	Song et al. (2017)
	Stathmin	An important soluble microtubule-assisted protein that has been highly conserved in sequence	Promote	Zhang et al. (2019)
	MMP-13	Matrix-metalloproteinase-13	Promote	Duncan et al. (2022)
	Berberine, Baicalein, and Wedelolactone	Herbal extracts	Promote	Lee et al. (2016), Wang et al. (2018), Wu et al. (2019)
	ELVs-H1	Exosome-like vesicles derived from a Hertwig’s epithelial root sheath (HERS) cell line	Promote	Zhang et al. (2020b)
	SATB2	Special AT-rich sequence-binding protein 2	Promote	Xin et al. (2021)
	Lead	Heavy metals ubiquitous in environment that accumulates in teeth and calcified tissues from where it releases gradually with aging and adversely affects dental health	Inhibit	Khalid et al. (2022)
Smad pathway	NFIC	Nuclear factor I-C	Promote	He et al. (2014)
	HtrA1	High-temperature requirement protein A1	Promote	Li et al. (2018a)
	MTA and GH	Mineral trioxide aggregate and growth hormone	Promote	Yun et al. (2016)
	Panx3	Pannexin3, a member of the pannexin gap junction protein family from tooth germs	Promote	Iwamoto et al. (2017)
	C2 ceramide	An activator of protein phosphatase 1 (PP1)	Promote	Kim et al. (2017)
	HERS-H1	An immortalized HERS cell line	Promote	Zhang et al. (2020a)
	Phenytoin	An antiseizure drug acting at the voltage-gated sodium channel	Promote	Shang and Xiong (2022)
	*P. corylifolia*	*Psoralea corylifolia L*, an Oriental phytomedicine	Promote	Jang et al. (2022)
	CKIP-1	Casein kinase 2 interacting protein-1	Inhibit	Song et al. (2019)
MAPK pathway	EREG	Epiregulin, a novel epidermal growth factor (EGF)-related growth regulating peptide	Promote	Cui et al. (2019)
	E2	Estrogen 17β-estradiol	Promote	Woo et al. (2015)
	RGD-3	Arginine-glycine-aspertic acid (RGD) peptide-3 from human dentin phosphophoryn (DPP)	Promote	Hassan et al. (2022)
	IGF	Insulin-like growth factor	Promote	He et al. (2022)
	Ca(OH)_2_	Calcium hydroxide	Promote	Chen et al. (2016b)
	Neu5Ac	N-acetylneuraminic acid, one form of sialic acid	Promote	Li et al. (2021)
	Simvastatin	a 3-hydroxy-3-methyl-glutaryl-coenzyme A reductase inhibitor, is a well-established cholesterol-lowering drug able to inhibit cholesterol synthesis	Promote	Karanxha et al. (2013)
	Wnt6	A member of Wnt family	Promote	Li et al. (2014)
	DLK1	Delta-like homologue 1, a transmembrane and secreted protein	Inhibit	Qi et al. (2017)
Notch pathway	Ca(OH)_2_	Calcium hydroxide	Promote	Lovschall et al. (2005)
	TN-C	Tenascin-C, a glycoprotein found in the ECM	Promote	Matsuoka et al. (2013)
	HBD4	Human β-defensin 4, one member of a family of small cationic polypeptides rich in cysteine	Promote	Zhai et al. (2019), Zhai et al. (2020)
	PRF	Platelet-rich fibrin	Promote	Zhang et al. (2022)
	Postn	Periostin, a 90 kDa secreted protein belonging to the fasciclin family	Inhibit	Zhou et al. (2015)

Nevertheless, there is still conflict regarding the role of Wnt/β-catenin signaling in odontoblast differentiation. Odontoblastic differentiation of hDPSCs can be enhanced by the long non-coding RNA (lncRNA) short nucleolar RNA host gene 1 (*SNHG1*), in which Wnt/β-catenin pathway is inhibited by microRNA-328-3p (miR-328-3p) (Fu et al., 2022).

In addition to the canonical Wnt/β-catenin signaling pathway, the non-canonical Wnt signaling pathway participates in the regulation of odontogenetic differentiation. Wnt5a, a typical non-canonical Wnt protein, is expressed in the dental papillary tissue and hDPCs (Peng et al., 2009; Lin et al., 2011b). In hDPCs, migration and proliferation are inhibited by Wnt5a (Peng et al., 2009), whereas differentiation is enhanced. Overexpression of Wnt5a upregulates alkaline phosphatase (ALP) activity and increases the expression of mineralization-related genes such as DMP1, osteopontin (OPN), bone sialoprotein (BSP), osteonectin (ON), and Col1 (Peng et al., 2010). In *Wnt5a* mutant mice, the expression of *Bmp4* and Msh (muscle segment homeobox) *Drosophila* homolog 1 (*Msx1*) is attenuated in the dental mesenchyme, and delayed odontoblastic differentiation is observed (Lin et al., 2011b). Although Wnt6 belongs to the canonical Wnt pathway, it has also been implicated in the non-canonical Wnt signaling pathway (Wei et al., 2020). Overexpression of Wnt6 promotes odontoblastic differentiation of human dental papilla cells with increased ALP activity; however, cell proliferation is hardly affected (Wang et al., 2010). These results demonstrate the role of non-canonical Wnt/β-catenin pathway on odontoblastic differentiation.

#### 3.1.2 Small mothers against decapentaplegia (Smad) signaling pathway

Smad proteins are the molecules that transmit signals of transforming growth factor β (TGF-β) and BMP family intracellularly. Smads are divided into three categories: receptor-regulated Smads (R-Smads), including Smad1, Smad2, Smad3, Smad5, Smad8, and Smad9; common-partner Smads (co-Smads); Smad4, which is associated with phosphorylated R-Smads; and inhibitory Smads (I-Smads), including Smad6 and Smad7 (Qin et al., 2012b).

##### 3.1.2.1 TGF-β/Smad signaling pathway

TGF family is multipotent in numerous biological processes (Hata and Chen, 2016). TGF-β superfamily is constitutive of TGF-βs, BMPs, Activin, Nodal etc. (Yu et al., 2021). Three transmembrane TGF-β receptors have been identified: the type I (TβRI), type II (TβRII), and type III (TβRIII) (Gilboa et al., 1998). TGF-β family members transduce signal across the membrane by interacting with TβRI and TβRII, moreover, TβRIII helps present ligand to the signaling receptors (Henis et al., 1994; Gilboa et al., 1998). TβRI consists of two subgroups, one group respond to TGF-β signals and phosphorylate Smad2 and Smad3, while the other group respond to BMP signals and phosphorylate Smad1, Smad5, and Smad8 (Hata and Chen, 2016). Once TβRIs are activated, they phosphorylate Smad proteins and initiate transcriptional responses in the nucleus (Chen et al., 2012; Hata and Chen, 2016). When TGF-β binds to TβRII, which is homomeric, then a heterotetrameric TβRI-TβRII complex is formed (Henis et al., 1994; Gilboa et al., 1998).

TGF-β/Smad signaling pathway is implicated in odontogenesis. Studies suggest that TGF-β/Smad pathway is present in odontoblasts (He et al., 2004). During tooth crown formation, TGF-β family is involved in odontoblast differentiation (Lesot et al., 2001). Smad signaling is activated in mouse dental papilla cell-23 (MDPC-23) by TGF-β1, as TGF-β1 activates Smad2, Smad3 and Smad4 (He et al., 2004). In DPCs cultured in scaffold material with TGF-β1, the ALP activity is increased, thus the odontoblastic differentiation is enhanced, and dentin formation is promoted (Li et al., 2011b). The levels of odontoblastic marker genes are increased by liposomal TGF-β1 in hDPSCs, including *DSPP*, *RUNX2*, and *DMP1*, which indicates that TGF-β1 positively induces odontoblastic differentiation of hDPSCs (Jiang et al., 2020). TGF-β1 inhibits and promotes the odontoblastic differentiation of SCAPs in the early and late stages (Yu et al., 2020). But TGF-β1 enhances early-stage odontogenic differentiation but diminishes later-stage mineralization in DPSCs (Bai et al., 2022). Odontoblastic differentiation of SCAPs is suggested to be promoted by TGF-β2. In SCAPs treated with TGF-β2, the expressions of odontoblastic markers are upregulated. The levels of odontoblastic markers are attenuated by knockdown of TGF-β2 *in vitro* and *in vivo*, including DSPP and DMP1 (Yu et al., 2020; Li et al., 2022a). TGF-β/Smad pathway is regulated by several factors (Figure 2; Table 1). The expression levels of odontogenic-related genes can be increased by overexpressing nuclear factor I-C (NFIC) via TGF-β1 stimulation in SCAPs (He et al., 2014). High-temperature requirement protein A1 (HtrA1) activates TGF-β1/Smad signaling pathway to promote the odontoblastic differentiation of hDPCs, as TGF-β1 and mRNA expression of downstream factor are increased (Li et al., 2018b).

Moreover, TGF-β signaling may negatively affect odontogenic differentiation. In MDPC-23 cells, the activity of the DSPP promoter is attenuated by TGF-β1 (He et al., 2004). In SCAPs treated with 2 ng/mL TGF-β1 for 2 weeks, the levels of ALP, DSPP, and OCN, decreased greatly. An *in vitro* study showed that TGF-β1 attenuated odontoblastic differentiation induced by Smad3. The negative role of TGF-β1 can be enhanced by NFIC knockdown (He et al., 2014). Odontoblastic differentiation is promoted when TGF-β1 is knocked down in SCAPs (Li et al., 2022a). Level of extracellular-signal regulated kinase1/2 (ERK1/2) and phosphorylation of Smad2/3 and Smad1/5/8 are increased by TGF-β2 in DPCs, whereas ALP activity is decreased. Therefore, TGF-β2 tends to inhibit DPCs differentiation via the ALK/Smad2/3 pathway (Tai et al., 2008).

However, Smad3, a regulator of TGF-β signaling, may play a negative regulatory role in odontoblastic differentiation. In MDPC-23 cells, the overexpression of Smad3 diminishes DSPP gene transcription by increasing the inhibitory ability of TGF-β1 (He et al., 2004). In contrast, ALP and OCN mRNA expression is upregulated in SCAPs treated with TGF-β1 and Smad3 inhibitors (He et al., 2014). The knockdown of Smad3 promotes the odontoblastic differentiation of DPSCs. As observed in mineralization-induced DPSCs, the knockdown of Smad3 induces the early expression of DSPP and DMP1, and ALP expression is increased (Huang et al., 2019).

##### 3.1.2.2 BMP/Smad signaling pathway

BMP signaling is also pivotal in dentin development. BMPs are members of the TGF-β superfamily (Hoodless et al., 1996). The Smad-dependent pathway is one of the pathways through which BMP regulate downstream gene expression. First, BMP ligands bind to type I (BMPR-I) serine/threonine kinases and type II receptors (BMPR-II). BMPR-I is then phosphorylated by BMPR-II, followed by the phosphorylation and activation of Smad1/5/8. Phosphorylated Smad1/5/8 heterodimers and Smad4 form a complex; consequently, BMP target genes are induced by nuclear translocation of this complex (Liu et al., 2022b). During dentinogenesis, the genes downstream of BMPs include *Runx2*, *Osx*, distal-less (Dlx) homeobox gene 3 (*Dlx3*), *Msx1*, *Msx2*, paired box 9 (*Pax9*), *DMP1*, and *DSPP* (Liu et al., 2022b).

The BMP/Smad signaling pathway is essential for odontoblastic differentiation. Smad1/5 can be activated by BMP2 as the phosphorylation and nuclear translocation of Smad1/5 are increased, and BMP signaling inhibitors attenuate odontoblastic differentiation and mineralization of DPCs. This suggests that BMP2 induces odontoblastic differentiation of DPCs via the Smad1/5 pathway (Qin et al., 2012b). In hDPCs treated with mineral trioxide aggregate (MTA) and growth hormone (GH), the levels of BMP2 mRNA and phosphorylation of Smad1/5/8, Runx2, and Osx were higher than those in the MTA-only control group, which suggests that MTA and GH positively mediate odontogenic differentiation via the BMP pathway (Yun et al., 2016). Odontoblastic differentiation of mDPCs is promoted by Pannexin3 (Panx3) via the BMP/Smad pathway. Following the overexpression of *Panx3*, phosphorylation of Smad1/5/8 induced by BMP2 and increased expression of *Dspp* were observed (Iwamoto et al., 2017). C2 ceramide, an activator of protein phosphatase 1 (PP1), leads to increased levels of BMP2 and phosphorylation of Smad 1/5/8, and enhances the ALP activity of hDPCs; therefore, odontogenic differentiation may be promoted through the BMP/Smad pathway (Kim et al., 2017). In DPCs, Smad4 and p-Smad1/5/8 are activated, and odontogenic markers are upregulated by HERS-H1. Therefore, odontoblastic differentiation is enhanced by HERS-H1 through the activation of the BMP/Smad signaling pathway (Zhang et al., 2020a). Phenytoin upregulates odontoblastic differentiation of hDPSC through the BMP4/Smad pathway and increases the expression of BMP4, Smad1/5/9, and p-Smad1/5 (Shang and Xiong, 2022). In hDPSCs treated with *Psoralea corylifolia L.* (*P. corylifolia*), an Oriental phytomedicine, the phosphorylation of Smad1/5/8 is upregulated, and odontoblastic differentiation was enhanced via the Smad signaling pathway (Jang et al., 2022). Inhibition of BMP/Smad pathway impairs dentin formation. Loss of *Bmp2* may cause dentinogenesis defects (Malik et al., 2018). Casein kinase 2 interacting protein-1 (CKIP-1) inhibits BMP2-Smad1/5 signaling, thus inhibiting the differentiation of DPSCs into odontoblasts (Song et al., 2019). These results demonstrated the critical role of the BMP/Smad signaling pathway.

##### 3.1.2.3 TGF-β superfamily/non-Smad signaling pathway

**FIGURE 3:**
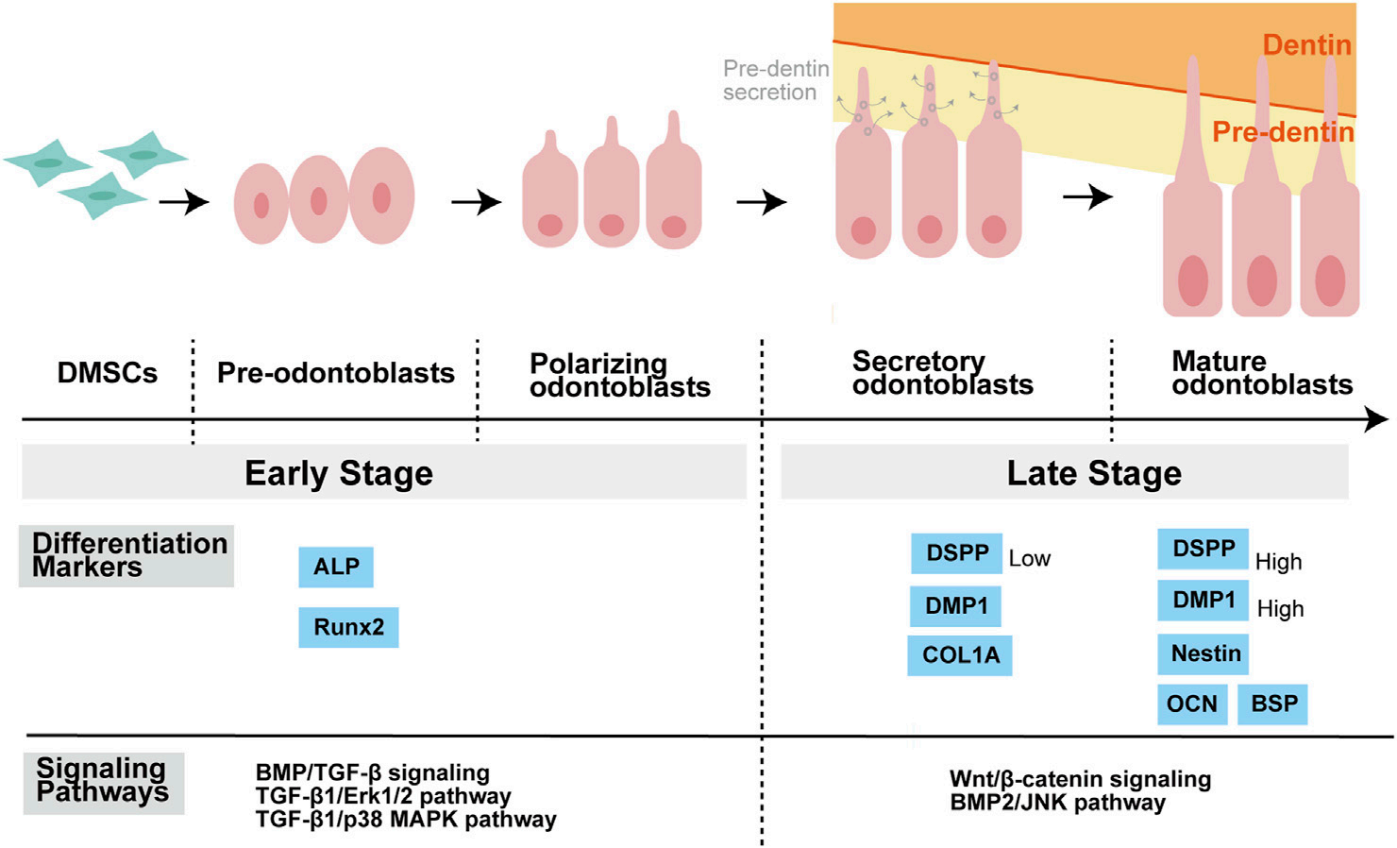
Stages of odontoblastic differentiation of DMSCs, and related differentiation markers as well as signaling pathways. DMSCs have the potential to differentiate into pre-odontoblasts, polarizing odontoblasts, secretory odontoblasts, and mature odontoblasts. DMSCs are spindle-like mesenchymal stem cells. Pre-odontoblasts are cells that stop dividing and increase in size than DMSCs, and whose organelles and cytoskeletal components are uniformly distributed in the cytoplasm. Polarization of pre-odontoblasts begins when they are going to differentiate into functional odontoblasts. During this process, the odontoblasts establish a cylindrical shape and exhibit structural polarity. Once polarized, odontoblasts differ in functional terms and are named secretory odontoblasts. Pre-dentine starts to be secreted at this stage. During the process of dentin formation, the odontoblast process is elongated gradually as a direct extension of the cell body. The matrix accumulates as unmineralized layer (pre-dentin) and gradually mineralizes to form dentin. ALP and Runx2 are known as the markers of the early stage of odontoblastic differentiation, while COL1A, DSPP, DMP1, OCN, BSP, and Nestin are regarded as the markers of the late stage of odontoblastic differentiation. Moreover, different signaling pathways may play a major role in different stages of differentiation. Balic, Anguila, and Mina stated that early stages of odontoblast differentiation include the stages of pre-odontoblasts and prior to the expression of Dmp1 and Dspp. For example, BMP/TGF-β signaling regulates odontoblast differentiation in the early stage of tooth formation. Activation of Erk1/2 and p38 MAPK pathways contributed to TGF-β1-induced early differentiation of DPSCs. While in the late stage of odontoblastic differentiation, Wnt signaling is important for terminal odontoblast differentiation, and Wnt/β-catenin signaling help pre-odontoblasts differentiate into functional and fully differentiation odontoblasts. And JNK is required for the late-stage differentiation of odontoblasts induced by BMP2. Abbreviations: DMSCs: Dental mesenchymal stem cells; ALP: alkaline phosphatase; Runx2: Runt-related transcription factor 2; COL1A: collagen type I A; DMP1: Dentine matrix protein 1; DSPP: Dentin sialophosphoprotein; OCN: Osteocalcin; BSP: Bone sialoprotein; BMP: Bone morphogenetic protein; TGF-β: Transforming growth factor β; ERK: Extracellular-signal regulated kinase; MAPK: Mitogen-activated protein kinase; JNK: c-Jun amino-terminal kinase.

Besides, non-Smad signaling pathways are also activated by TGF-β superfamily members in the process of odontoblastic differentiation. In hDPCs, BMP2 activates p38a MAPK as it induces the phosphorylation of p38α depending on dose and time, and p38 MAPK pathway regulates the stimulation of BMP2 (Qin et al., 2012a; Yang et al., 2015). Qin et al. first demonstrated that in BMP2-induced DPCs, JNK MAPK is specifically implicated in the late-stage of odontoblastic differentiation (Qin et al., 2014). While in the early phase of odontoblastic differentiation in DPSCs, TGF-β1 enhances odontoblastic differentiation via AKT, Erk1/2 and p38 MAPK signaling pathways, instead of Smad3 or JNK pathways (Bai et al., 2023) (Figure 3).

#### 3.1.3 Mitogen-activated protein kinase (MAPK) signaling pathway

**FIGURE 4:**
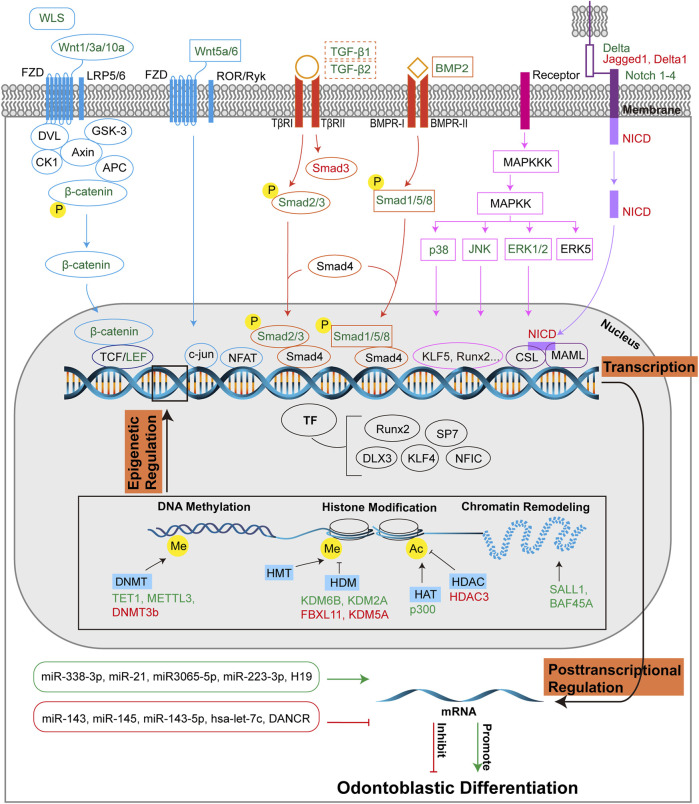
Molecular regulation of odontoblastic differentiation. Odontoblastic differentiation of DMSCs is modulated by transcriptional, post-transcriptional and epigenetic regulations. At the transcriptional level, signaling pathways include Wnt, Smad, MAPK, and Notch pathways, and corresponding transcriptional factors are depicted in the figure. The four signaling pathways are colored in blue, orange, pink, and purple, respectively, which are consistent with those in Figure 2. The signaling molecules or receptors in green suggest their positive role in odontoblastic differentiation, while the signaling molecules or receptors in red suggest their negative role in odontoblastic differentiation. The dotted-lined box indicates the dual role of the signaling factors in odontoblastic differentiation. At the posttranscriptional level, miRNAs and lncRNAs are positively (green box, green arrow) or negatively (red box, red T-shaped arrow) implicated in odontoblastic differentiation. Epigenetic regulations include DNA methylation, histone modification, and chromatin remodeling. DNMTs, HDMs, HAT, HDAC, and molecules associated with chromatin remodeling promote (green) or inhibit (red) odontoblastic differentiation at the epigenetic level of regulation. Abbreviations: WLS: Wntless; FZD: Frizzled receptor; LRP5/6: LDL receptor-related protein 5/6; DVL: Dishevelled; GSK-3: Glycogen synthase kinase 3; CK1: Casein kinase 1; Axin: Axis inhibition protein; APC: Adenomatous polyposis coli; TCF/LEF: T-cell-specific factor/lymphoid enhancer-binding factor; ROR/RYK: Retinoic acid related orphan receptor (ROR)/receptor tyrosine kinase (RYK); NFAT: Nuclear factor of activated T cells; Smad: Small mothers against decapentaplegia; TGF-β: Transforming growth factor β; TβR: TGF-β receptor; BMP: Bone morphogenetic protein; BMPR: BMP receptor; MAPK: Mitogen-activated protein kinase; MAPKK: MAP kinase kinase; MAPKKK: MAP kinase kinase kinase; ERK: Extracellular-signal regulated kinase; JNK: c-Jun amino-terminal kinase; Notch: Neurogenic locus notch homolog; Delta: DLL-type ligands; Jagged: JAG-type ligand; NICD: Notch intracellular domain; CSL: CBF1/Suppressor of hairless/Longevity-assurance gene-1; MAML: Mastermind-like protein; TF: Transcriptional factor; Runx2: Runt-related transcription factor 2; SP7: Specificity protein 7; DLX3: Homeobox gene distal-less 3; KLF: Krüppel-like factor; NFIC: Nuclear factor I-C; DNMT: DNA methyltransferase; TET1: ten-eleven translocation 1; METTL3: N6-methyladenosine (m^6^ A) methyltransferase; HMT: Histone methyltransferase; HDM: Histone demethylase; KDM: Histone demethylase lysine(K)-specific demethylase; FBXL11: KDM2A; HAT: Histone acetyltransferase; HDAC: Histone deacetylase; Me: Methyl; Ac: Acetyl; miR: microRNA; H19: lncRNA-H19; DANCR: Differentiation antagonizing non-protein coding RNA.

The MAPK pathway controls various physiological processes, including cell proliferation, gene expression, and apoptosis. This is mediated by ERK, c-Jun amino-terminal kinase (JNK), and p38 protein kinases (Johnson and Lapadat, 2002). ERK controls mitosis, JNK regulates transcription and inflammatory cytokines, and environmental stress may activate p38 (Johnson and Lapadat, 2002). MAPK pathways can be divided into conventional and atypical pathways. The conventional MAPK pathway consists of the classical cascade of MAP kinase kinase kinase (MAPKKK), MAP kinase kinase (MAPKK or MEK), and MAPK, in which MAPK is the effector-phosphorylating substrate. Typical MAPKs, including p38s, ERK1/ERK2, JNKs, and ERK5, possess a distinct Thr-Xaa-Tyr motif in the activation loop and thus can be activated by MAPKKs. However, atypical MAPK pathway lacks the three-tiered MAPKKK-MAPKK-MAPK cascade, and related MAPKs are absent of the Thr-Xaa-Tyr motif, such as ERK3/4, ERK7, and Nemo-like kinase (NLK) (Coulombe and Meloche, 2007). In this review, we summarize the roles of the three best-known conventional MAPK pathways in odontoblastic differentiation (Figure 4).

The p38 MAPK pathway is involved in odontoblastic differentiation. Activated p38a MAPK phosphorylates downstream TFs to initiate odontoblastic gene expression and hDPC differentiation, whereas blockage of p38α MAPK inhibits odontoblastic differentiation (Qin et al., 2012a). In MDPC-23 cells, ALP activity and osteogenic markers were reduced after blocking the p38-MAPK signaling pathway (Tang and Saito, 2018). Inhibition of the MAPK pathway also reduces odontoblastic differentiation of hDPSCs (Gu et al., 2022). Moreover, various factors function positively via the p38 MAPK pathway to promote odontoblastic differentiation as shown in Figure 2 and Table 1. Epiregulin (EREG) upregulates odontoblastic differentiation of DPSCs by increasing the phosphorylation of p38 MAPK (Cui et al., 2019). In hDPCs, Estrogen 17β-estradiol (E2) increases the expression of odontoblastic differentiation markers, enhancing odontoblastic differentiation by activating c-Src and MAPKs (Woo et al., 2015). The arginine-glycine-aspertic acid (RGD) peptide RGD-3 mediates odontogenic-related gene expression and enhances odontoblastic differentiation, during which the p38 MAPK pathway is activated in DPSCs (Hassan et al., 2022). The insulin-like growth factor (IGF) family contributes to odontoblast differentiation via the MAPK pathway (He et al., 2022). These results indicate the significance of the p38 MAPK pathway in odontoblastic differentiation.

The ERK pathway is critical for the promotion of odontoblastic differentiation. The MEK/ERK pathway contributes to the activation and phosphorylation of RUNX2 (Xiao et al., 2002), a pivotal transcription factor (TF) related in odontoblastic differentiation. When the ERK signaling pathway was blocked by the specific inhibitor U0126, the levels of odontoblastic markers were significantly downregulated (Karanxha et al., 2013). Moreover, it is associated with various factors involved in odontogenesis regulation as shown in Figure 2 and Table 1. For instance, in hDPCs, the ERK pathway functions positively during simvastatin-induced odontoblastic differentiation (Karanxha et al., 2013). Mechanical stress promotes odontoblastic differentiation of SCAPs through the ERK and JNK pathways, where the protein levels of pERK and pJNK are upregulated (Mu et al., 2014). Calcium hydroxide (Ca(OH)_2_) enhances DPSC differentiation by promoting the expression of p38, JNK, and ERK (Chen et al., 2016b). EREG also enhances ERK1/2 phosphorylation to increase odontoblastic differentiation of DPSCs (Cui et al., 2019). N-acetylneuraminic acid (Neu5Ac) promotes odontoblastic differentiation of DPSCs by activating the ERK pathway (Li et al., 2021). However, odontoblastic differentiation of hDPSCs is diminished by delta-like homolog 1 (DLK1) via the ERK pathway (Qi et al., 2017).

The JNK pathway has also been implicated in odontoblast differentiation. In hDPCs, Wnt6 enhances the JNK signaling pathway, thus increasing cell migration and differentiation; however, inhibition of the JNK pathway reduces Wnt6-induced odontoblastic differentiation (Li et al., 2014). In contrast, the suppression of JNK MAPK attenuates the odontoblastic differentiation of DPCs in the late phase (Qin et al., 2014) (Figure 3).

#### 3.1.4 Neurogenic locus notch homolog (Notch) signaling pathway

Notch signaling is involved in cell differentiation, proliferation, and death (Cai et al., 2011). Four Notch pathway receptors have been identified: Notch1, Notch2, Notch3, and Notch4. Notch ligands are divided into two categories: typical Notch ligand, including DLL-type ligands (Delta-like1, Delta-like3 and Delta-like4) and JAG-type ligands (Jagged1, Jagged2), and atypical Notch ligands, including DNER, F3⁄Contactin, and NB-3 (Eiraku et al., 2002; Cai et al., 2011). Compared to atypical ligands, typical ligands have a conserved DSL domain and higher affinity (Cai et al., 2011). When Notch ligands bind to their receptors, metalloprotease TNF-a converting enzyme and c-Secretase complex may cut the S2 and S3 sites of the receptors, and the Notch intracellular domain (NICD) is formed. NICD transmits signals to the nucleus and induces gene transcription (Cai et al., 2011). Notch signaling consists of the canonical and non-canonical Notch pathways. The canonical Notch pathway can be briefly identified as an NICD-CSL-MAML cascade activated by interactions between typical Notch ligands and receptors. Non-canonical Notch pathway is not well known, but it has different target genes and mediators than the canonical pathways (Cai et al., 2011).

The Notch signaling pathway is also involved in odontoblastic differentiation (Figure 4). Notch receptors and ligands are found in the dental epithelium or mesenchyme during odontogenesis, suggesting that Notch signaling may be involved in dentin formation (Zhang et al., 2008). *In vitro* study demonstrates that Notch2-Delta signaling positively mediates the odontoblastic differentiation of DPCs (He et al., 2003). The Notch pathway is activated by various stimulants, as shown in Figure 2 and Table 1. *In vivo* study showed that the Notch pathway was activated after pulp capping with Ca(OH)_2_, as increased expression of *Notch1*, *Notch2*, *Notch3*, *Delta1*, *Jagged1* and *Hes1* was observed. This suggests that pulp capping with Ca(OH)_2_ promotes odontoblastic differentiation of DPCs (Løvschall et al., 2005). Notch1, Notch2, ALP, OPN, and OCN mRNAs are upregulated in rat DPCs treated with tenascin-C (TN-C), suggesting that TN-C induces odontogenic differentiation through the Notch pathway (Matsuoka et al., 2013). Human β-defensin 4 (HBD4) increases odontoblast differentiation of SHEDs and DPSCs through the Notch pathway; therefore, HBD4 may be a prospective agent for pulp capping (Zhai et al., 2019; Zhai et al., 2020). Platelet-rich fibrin (PRF) increases the expression of important Notch signaling proteins such as Notch1, Jagged1, and Hes1, and upregulates odontoblastic markers in hDPSCs. Thus, PRF enhances odontoblastic differentiation (Zhang et al., 2022). Overexpression of periostin (Postn) inhibited odontoblast differentiation of mDPCs, and downregulation of Notch signaling molecules was observed. Therefore, downregulation of the Notch pathway adversely affects odontoblastic differentiation (Zhou et al., 2015). These results demonstrate the positive role of the Notch pathway.

However, Notch signaling can negatively affect odontoblastic differentiation. Zhang et al. first demonstrated that overexpressed Jagged1 activates the Notch pathway; consequently, odontoblastic differentiation of DPSCs is decreased *in vitro* and *in vivo* (Zhang et al., 2008). Overexpression of NICD diminishes the odontoblastic differentiation of DPSCs, suggesting a negative effect on the Notch pathway. DSPP expression is downregulated by Notch signaling, and mechanistically, Notch signaling may inhibit Runx2-dependent gene transcription via the Notch target gene *Hes1* (Zhang et al., 2008). Moreover, hDPSCs with inhibition of Delta1 tend to differentiate into odontoblasts compared with the control group (Wang et al., 2011).

### 3.2 Transcriptional factors (TFs)

TFs are important for many physiological processes. Different signaling pathways are linked by TFs, which subsequently initiate specific gene expression by binding to enhancers and promoters (Tao et al., 2019). Recently, TFs were reported to be involved in dentinogenesis. Frequently mentioned odontoblast-related TFs are summarized in this review, including RUNX2, SP7, DLX3, KLF4, NFIC etc. (Figure 4).

#### 3.2.1 Runt-related transcription factor 2 (RUNX2)

RUNX2 is a well-known TF belonging to the *Drosophila* Runt family, which regulates osteo/odontoblast differentiation (Li et al., 2011a). RUNX2 plays an important role in odontoblastic differentiation (Camilleri and McDonald, 2006; Han et al., 2014). *In vivo* study showed that knockout of *Runx2* leads to defective odontoblastic differentiation during the early phase of odontogenesis (D'Souza et al., 1999). However, forced overexpression of Runx2 interfered with late-stage odontoblast differentiation *in vivo* (Li et al., 2011a).

#### 3.2.2 Specificity protein 7 (SP7)

Sp7, also known as Osx, belongs to the zinc finger-containing Sp1 family of TFs (Bae et al., 2018), and is critical for enhancing odontoblastic differentiation. Sp7 is expressed in undifferentiated ectomesenchymal cells and odontoblasts, and is a downstream target of Runx2 (Komori et al., 1997; D’Souza et al., 1999; Hosoya et al., 2013; Tomazelli et al., 2015). The expression of odontoblastic markers, including *Alp*, *Dspp*, *Dmp1*, and *nestin*, was significantly increased in human dental papilla cells overexpressing Sp7 (Yang et al., 2014). Mechanistically, odontoblastic-related genes are directly mediated by Sp7, including *ALP*, *Dspp* and *Ocn* (Yang et al., 2014; Bae et al., 2018). However, an *in vivo* study demonstrated that *Sp7* knockout impairs odontoblastic differentiation (Bae et al., 2018).

#### 3.2.3 Homeobox gene distal-less 3 (DLX3)

DLX3 is a member of the DLX family and is involved in tooth development (Ghoul-Mazgar et al., 2005). During odontoblast differentiation, Dlx3 expression increases at mRNA and protein levels (Li et al., 2012). In hDPCs, Dlx3 upregulated ALP activity and DSPP and DMP1 levels. Therefore, Dlx3 enhances odontoblastic differentiation of hDPCs (Li et al., 2012). In a mouse model knockout of *Dlx3* in the neural crest, defective odontoblast differentiation and impaired dentin formation were observed, and DSPP levels decreased significantly (Duverger et al., 2012). Dlx3 directly regulates Oc and Runx2 in *ex vivo* studies on bone (Duverger et al., 2012), and Runx2 is a critical TF in odontoblastic differentiation.

#### 3.2.4 Krüppel-like factor 4 (KLF4)

KLF4 is homogenic to the *Drosophila melanogaster* Krüppel protein and is pivotal during odontoblastic differentiation (Tao et al., 2019). Expression of KLF4 been observed in the mouse polarizing odontoblast layer, suggesting that KLF4 may positively affect odontoblastic differentiation at the terminal stage (Chen et al., 2009b). In hDPCs, overexpression of KLF4 results in increased levels of ALP and the odontogenic markers DMP1 and DSPP (Lin et al., 2011a). Mechanistically, KLF4 promotes odontoblastic differentiation by activating TGF-β signaling pathway in the initial stage, in which *Runx2* is a cofactor. KLF4 also increases the transcription of *Dmp1* and *Sp7* by binding to their promoters and regulating histone acetylation. KLF4 interacts with HDAC3 and P300 during the early and later stages of odontoblastic differentiation, respectively (Lin et al., 2013; Tao et al., 2019). Enlarged pulp canals and dentin mineralization defects have been observed in *Klf4*-knockout mouse models (Tao et al., 2019). When *Klf4* transcription is activated by the binding of NFIC through binding to its promoter, the expression of *Dmp1* and *DSPP* is elevated, thus promoting odontoblast differentiation (Lee et al., 2014). In conclusion, KLF4 promotes odontoblastic differentiation.

#### 3.2.5 NFIC

NFIC belongs to the nuclear factor I family, which contains three other members: NFIA, NFIB, and NFIX (Gronostajski, 2000). NFIC enhances odontoblastic differentiation of SCAPs. *In vitro* studies have suggested that up- and downregulation of NFIC promotes and inhibits the levels of odontoblast-related markers, including ALP, Col1, and OCN (Zhang et al., 2015b). NFIC-extracellular vesicles (EV), which deliver NFIC to SCAPs, enhance odontoblastic differentiation by upregulating ALP activity and odontoblastic markers, such as ALP, DSPP, and DMP1. Additionally, dentin formation can be promoted by NFIC-EVs *in vivo* (Yang et al., 2022). While in *Nfic* ^−/−^ mice models, some characteristic odontoblastic markers are reduced, including DSPP, DMP1, OCN, Col 1, and nestin (Xu et al., 2022). These results suggested that NFIC is important for odontoblastic differentiation.

#### 3.2.6 Others

In addition to the TFs mentioned above, many other TFs participate in the regulation of odontoblastic differentiation in DMSCs. In the mouse immortalized dental papilla mesenchymal cell line (iMDP-3), overexpression of Klf5, Klf6, and Klf10 promotes odontoblastic differentiation. Mechanistically, the transcription of *Dspp* and *Dmp1* is enhanced by Klf5, Klf6, and Klf10 (Chen et al., 2016c; Chen et al., 2017; Chen et al., 2021). ATF6, an endoplasmic reticulum (ER) membrane-bound TF, is involved in hDPC odontoblastic differentiation. Overexpression of ATF6 results in an increased expression of DSPP and DMP1 (Kim et al., 2014). The expression of zinc finger E-box-binding homeobox 1 (*Zeb1*) is observed in the tooth germ mesenchyme and increases during odontogenic differentiation *in vivo* and *in vitro*. When *Zeb1* is inhibited, the differentiation of mDPCs is therefore reduced. Mechanistically, the expression of Runx2 and Dspp is promoted by ZEB1 respectively in the early and late phases of odontoblastic differentiation (Xiao et al., 2021). BTB and CNC homology 1 (BACH1) is a transcription repressor present in the odontoblast layer. Upregulation and downregulation of BACH1 induces odontoblastic differentiation in a positive and negative manner, respectively, by interacting with HO-1 (Liu et al., 2022a). Hypoxia-inducible factor 1 (HIF1) is a TF activated by hypoxic circumstances, whose subunit HIF1α proves to enhance odontoblast differentiation. In SHEDs from patients with fibrodysplasia ossificans progressiva, BMP pathway is activated by HIF1α, thus odontoblast differentiation is promoted (Wang et al., 2016). Moreover, HIF1α upregulates odontogenic differentiation of hDPSCs via Wnt/β-catenin pathway synergically with BCL9 (Orikasa et al., 2022).

### 3.3 Posttranscriptional regulation

In the process of odontoblastic differentiation, posttranscriptional regulation is involved in odontoblastic differentiation. This review provides an overview of the regulation by microRNAs (miRNAs, miRs) and long noncoding RNAs (lncRNAs) at the posttranscriptional level.

#### 3.3.1 miRNAs

MiRNAs, a subgroup of endogenous ∼22-nucleotide-long noncoding RNA, have been implicated in posttranscriptional gene regulation (Ambros, 2004; Sun et al., 2015). miRNAs interact with target mRNAs by binding to the 3′ untranslated region (3′UTR) to arrest translation and attenuate protein expression (Yue et al., 2012).

miRNAs, including miR-338-3p, miR-21, miR-3065-5p, and miR-223-3p, can positively mediate odontoblastic differentiation, as shown in Figure 4. miR-338-3p targets Runx2 to enhance the terminal differentiation of odontoblasts (Sun et al., 2013). In hDPSCs treated with low concentration of tumor necrosis factor-α (TNF-α), the levels of miR-21 and transducer and activator of transcription 3 (STAT3) increased, suggesting the positive function of miR-21/STAT3 signal in the regulation of odontoblastic differentiation (Xu et al., 2018). The level of miR-3065-5p increases during odontoblastic differentiation. It binds to the 3′-UTR of BMPR-II to promote odontoblastic differentiation (Lin et al., 2018). miR-223-3p targets to inhibit Smad3, which is an intracellular effector of TGF-β signaling pathway, therefore enhances odontoblastic differentiation of hDPSCs (Huang et al., 2019).

However, some miRNAs, such as miR-143, miR145, miR-143-5p, and hsa-let-7c, inhibit odontoblastic differentiation, as shown in Figure 4 (Fang et al., 2019). In the mDPC6T cell line, downregulation of miR-143 and miR-145 led to elevated expression of *Dspp* and *Dmp1*, indicating enhanced odontoblastic differentiation (Liu et al., 2013). Downregulation of miR-143-5p elevates the expression of *MAPK14* by arresting the binding to the *MAPK14* 3′-UTR, thus p38 MAPK pathway is activated to enhance odontoblastic differentiation of hDPSCs (Wang et al., 2019). In SCAPs treated with IGF-1, low expression of hsa-let-7c promoted the expression of odontoblastic markers. Mechanistically, hsa-let-7c targets IGF-1R and the IGF-1/IGF-1R/hsa-let-7c axis regulates odontoblastic differentiation (Ma et al., 2016).

However, there are conflicting results regarding the function of miR-140-5p in odontoblastic differentiation. It has been demonstrated that miR-140-5p enhances odontoblastic differentiation of DPSCs through Wnt1/β-catenin signaling (Lu et al., 2019). Overexpression of miR-140-5p can downregulate DPSC differentiation, and *vice versa*, in which miR-140-5p binds to the 3′-UTR of TLR4 mRNA to modulate TLR4 (Sun et al., 2017; Zhong et al., 2020).

#### 3.3.2 lncRNA

LncRNAs are a class of ncRNAs with a length of more than 200 nucleotides (Fang et al., 2019). lncRNAs can modulate gene expression at the transcriptional, posttranscriptional, and epigenetic levels (Wu et al., 2017; Kopp and Mendell, 2018). Recent studies have found that some lncRNAs, including lncRNA-H19 (H19) and differentiation-antagonizing non-protein coding RNA (DANCR), transcriptionally regulate odontoblastic differentiation, as shown in Figure 4.

H19 promotes odontoblastic differentiation at the posttranscriptional level. During odontoblastic differentiation of hDPSCs, notably increased expression of H19 has been observed (Zhong et al., 2020). *In vitro* and *in vivo* studies suggest that odontoblastic differentiation of hDPSCs and SCAPs can be enhanced by the overexpression of H19 but inhibited by its downregulation (Li et al., 2019; Zhong et al., 2020). H19 promotes the odontoblastic differentiation of hDPSCs via the H19/SAHH axis (Zeng et al., 2018a). The odontoblastic differentiation of SCAPs is promoted by H19 via the miR-141/SPAG9 pathway (Li et al., 2019). Mechanistically, H19 interacts with miRNAs. For instance, H19 stops the miRNA-mediated degradation of SPAG9 by competitively binding to miR-141; therefore, the phosphorylation of p38 and JNK is increased and the differentiation of SCAPs is promoted (Li et al., 2019). Moreover, H19 attenuates miR-140-5p′s inhibitory activity on odontoblastic differentiation by acting as a sponge for miR-140-5p. Therefore, H19 promotes the expression of BMP2 and FGF9, and enhances hDPSCs differentiation into odontoblasts (Zhong et al., 2020). These results demonstrated the positive function of H19 in the regulation of odontoblastic differentiation.

However, the lncRNA DANCR has a negative impact on odontoblastic differentiation. Kretz et al. first identified an lncRNA and named it anti-differentiation non-coding RNA (ANCR), and subsequently named it DANCR (Kretz et al., 2012). The expression of DANCR decreases during odontoblastic differentiation of hDPCs in a time-dependent manner (Chen et al., 2016a), indicating that DANCR may be negative for hDPCs differentiation. In hDPCs overexpressing DANCR, the levels of odontogenic markers, such as DSPP and DMP1, are downregulated. And upregulation of DANCR leads to lowered expression of phosphorylation of GSK3β (p-GSK3β) and β-catenin, suggesting the inhibition of Wnt/β-catenin signal pathway and odontoblastic differentiation (Chen et al., 2016a). Moreover, DANCR acts as a sponge of miR-216a by directly binding to it. Therefore, DANCR sponges miR-216a to inhibit the odontoblastic differentiation of hDPCs by enhancing the expression of c-CBL, which suppresses odontoblastic differentiation but can be inhibited by miR-216a (Chen et al., 2020). These results suggest a negative role for DANCR in odontoblastic differentiation.

Recent studies have identified other lncRNAs involved in odontoblastic differentiation. Tu et al. identified a novel lncRNA named CALB2, which enhances the odontoblastic differentiation of hDPCs via the CALB2/miR-30b-3p/RUNX2 axis by sponging miR-30b-3p and upregulating RUNX2 (Tu et al., 2020). The lncRNA IGF-binding protein 7-antisense 1 (IGFBP7-AS1) positively mediates the odontogenetic differentiation of SHEDs through the p38 MAPK pathway (Wang et al., 2022), which can be negatively regulated by miR-335-3p and miR-155-5p (Zhu et al., 2022). Under hypoxic conditions, the odontoblastic differentiation activity of DPSCs is attenuated. Six-twelve leukemia (STL) were identified by bioinformatics analysis as candidate lncRNAs associated with DPSCs. STL knockdown inhibits odontoblastic differentiation possibly by regulating NQO1 (nicotinamide adenine dinucleotide (NADH): quinone oxidoreductase 1) and ERO1 (endoplasmic reticulum oxidoreductin 1) (Shi et al., 2019).

### 3.4 Epigenetic regulation

Epigenetic regulation, including DNA methylation and histone tail modification, has recently been shown to modulate odontoblastic differentiation, as shown in Figure 4 (Kamiunten et al., 2015).

#### 3.4.1 DNA methylation

DNA methyltransferases (DNMTs), including DNMT1, DNMT3A, and DNMT3B, regulate DNA methylation, which tends to silence the promoter and enhancer classes (Smith and Meissner, 2013). DNA methylation is an important epigenetic regulator of odontoblast differentiation. The loss of ten-eleven translocation 1 (TET1), a DNA methyl cytosine dioxygenase, arrests hydroxymethylation and transcription of the Family with Sequence Similarity 20C (FAM20C), thereby inhibiting odontoblastic differentiation of hDPCs (Li et al., 2018a). In DPSCs, the H19/SAHH axis enhances odontoblastic differentiation by diminishing the methylation of DLX3 mediated by DNMT3B (Zeng et al., 2018a). In pre-odontoblastic cells, loss of DNMTs promotes odontoblastic differentiation by elevating the expression of *Klf4* and odontoblastic marker genes. SP1 modulates *KLF4* via a demethylated binding site on a CpG island in *KLF4* promoter region (Sun et al., 2019). N6-methyladenosine (m^6^ A) methyltransferase METTL3 promotes odontoblastic differentiation. Absence of *METTL3* in hDPCs inhibits *NFIC* translation. Consequently, the knockdown of *METTL3* results in decreased odontogenic differentiation *in vitro*, and reduced dentin formation in the root has been observed *in vivo* (Sheng et al., 2021). These results demonstrate the dual role of DNA methylation in odontoblastic differentiation.

#### 3.4.2 Histone modification

Epigenetic modifications of histones include methylation, acetylation, phosphorylation, and ubiquitylation (Chen and Dent, 2014), among which methylation and acetylation are the most frequently mentioned histone modifications that are involved in odontoblastic differentiation.

##### 3.4.2.1 Histone methylation

Histone methyltransferases (HMTs) and demethylases (HDMs) control histone methylation, which is the epigenetic modulation critical in many psychological processes (Du et al., 2013; Li et al., 2020).

HMTs are involved in odontogenesis. The four histone 3 lysine 9 (H3K9) MTs, G9a, GLP, PRDM2, and SUV39H1, were expressed abundantly in the tooth germ, and their expression reached a peak at E16.5 and E17.5. This suggests that the four H3K9MTs regulate tooth development and cell differentiation (Kamiunten et al., 2015).

Histone demethylase lysine(K)-specific demethylases (KDMs) tend to induce odontoblastic differentiation. KDM6B binds to the BMP2 promoter and activates odontogenic transcriptional genes by removing trimethylated H3K27 (H3K27me3). In DMSCs, knockdown and overexpression of KDM6B result in the attenuation and upregulation of odontoblastic differentiation, respectively (Xu et al., 2013). In SCAPs, the H3K4 demethylase KDM2A induces cell proliferation by upregulating p15 (INK4B) and p27 (Kip1) (Gao et al., 2013).

However, HDMs and KDMs sometimes suppress odontoblastic differentiation. For instance, FBXL11 (KDM2A) binds to the BCL6 co-repressor for activation; consequently, *EREG* transcription is inhibited by the increased methylation of histone K4/36 in the *EREG* promoter. Therefore, FBXL11 inhibits odontoblastic differentiation of SCAPs (Du et al., 2013). KDM5A negatively modulates the odontoblastic differentiation of hDPCs by deleting H3K4me3 from the promoters of target genes, and the inhibition of KDM5A increases H3K4me3 levels, as well as ALP activity and odontogenic markers (Li et al., 2020). Inhibition of HDMs leads to the upregulation of H3K4me3 and promotes odontoblastic differentiation (Yuan et al., 2022).

##### 3.4.2.2 Histone acetylation

Histone acetyltransferases (HATs) and deacetylases (HDACs) are responsible for histone acetylation, which changes the interaction between histone proteins, DNA, and nuclear proteins, and serves as a type of epigenetic regulation during odontoblastic differentiation (Tao et al., 2020).

Histone acetylation positively regulates odontoblastic differentiation. Overexpression of HAT p300 in DPSCs cultured for odontoblastic differentiation results in an increase in the expression of odontogenic marker genes, including *DMP1*, *DSPP*, *DSP*, *OPN* and *OCN* (Wang et al., 2014b). Acetylation of histone H3 lysine 9 (H3K9ac) and H3K27ac is elevated during odontoblast differentiation, with an increase in p300 and a decrease in HDAC3 (Tao et al., 2020). *In vitro* study demonstrated that the upregulation of HDAC3 or the loss of p300 negatively mediated odontoblast differentiation (Tao et al., 2020). HDAC inhibitors, including trichostatin A (TSA), MS-275, and LMK-235, enhance the odontoblastic differentiation of DPSCs (Jin et al., 2013; Liu et al., 2018b; Lee et al., 2020; Tao et al., 2020). The inhibition of HDACs leads to increased H3K27ac levels, thereby promoting odontogenic differentiation (Yuan et al., 2022). These results indicate a positive effect of histone acetylation on odontoblastic differentiation.

#### 3.4.3 Chromatin remodeling

Chromatin remodeling is a less-studied part of epigenetics but is also associated with odontogenic differentiation. ATP-dependent enzymes remodel chromatin and are important for modulating chromatin structure and assembly (Ho and Crabtree, 2010). SALL1, which is expressed in pre-odontoblasts *in vivo*, promotes the odontoblastic differentiation of mouse dental papilla cells by activating cis-regulatory elements near *Tgf-β2* and within the *Runx2* locus to remodel open chromatin regions (Lin et al., 2021). *Baf45a* belongs to the ATPase-dependent switching defective/sucrose non-fermenting (SWI/SNF) chromatin remodeling complex. Knockdown of *Baf45a* leads to the downregulation of TFs that regulate odontoblast differentiation-related marker genes. It has been demonstrated that BAF45A induces remodeling of the promoters of genes that promote odontoblast differentiation in a transcriptional manner (Busby et al., 2021).

## 4 Genetic syndromes with dentin defects caused by impaired odontoblast differentiation

### 4.1 Tricho-dento-osseous (TDO) syndrome

#### 4.1.1 Etiology

TDO syndrome (Online Mendelian Human Genetics (OMIM) database 190320) is caused by *DLX3* mutations, and the c.571_574delGGGG mutation in *DLX3* (MT-DLX3) is the most common etiologic mutation of TDO (Price et al., 1998a; Price et al., 1998b).

Choi et al. developed transgenic mice expressing MT-DLX3 and observed evident dentin defects and enlarged unmineralized pulp in patients with TDO. MT-DLX3 has been demonstrated to affect odontoblastic differentiation, resulting in increased odontoblast apoptosis and distortion of dentin tubule production and dentin matrix formation, thereby downregulating dentin formation and taurodontism appeared (Choi et al., 2010).

A recently identified mutation (c.533 A > G; Q178R) in *DLX3* (MU-DLX3) was reported to cause TDO syndrome (Li et al., 2015). MU-DLX3 decreases the proliferation rate and restrains odontoblastic differentiation and mineralization of hDPCs, consequently impairing dentinogenesis (Zeng et al., 2017). miR-675 enhances and facilitates odontoblastic differentiation of hDPCs by regulating DLX3 at the epigenetic level by inhibiting DNMT3B-mediated methylation of DLX3 (Zeng et al., 2018b). Downregulated expression of lncRNAs H19 and its co-expression product, miR-675, can be observed in TDO patients with *DLX3* mutations (Zhao et al., 2017). MU-DLX3 largely diminishes hDPCs differentiation through the H19/miR-675 axis and changes the expression and methylation of H19 by upregulating H3K9me3 accumulation and DNMT3B activity, leading to dentin hypoplasia (Zeng et al., 2019).

#### 4.1.2 Clinical features

##### 4.1.2.1 Systematic features

TDO is an autosomal dominant (AD) condition characterized by anomalies in hair, teeth, and bones. TDO patients have kinky, curly hair which is featured and distinguished (Wright et al., 1997). Patients suffer from obliteration of the diploe and a lack of visible mastoid pneumatization (Price et al., 1998b). Bone density increases in the long bones, vault, base of the skull, and mastoid process, which may result from cortical sclerosis (Crawford and Aldred, 1990; Islam et al., 2005). Dolichocephaly, caused by the early closure of cranial sutures and shortened mandibles, is also a skeletal feature of TDO (Crawford and Aldred, 1990). However, phenotypic heterogeneity exists among TDO patients, which may be due to environmental or genetic factors (Jain et al., 2017). For example, curly hair at birth and dental defects such as taurodontism and enamel hypoplasia may vary from person to person clinically (Wright et al., 1997).

##### 4.1.2.2 Dental features

Dental features including thin enamel, thin dentin, and taurodontism may be the most distinct characteristics of TDO patients (Wright et al., 1994), including thin enamel, thin dentin, and taurodontism. The teeth of patients with TDO exhibit generalized thin and/or pitted enamel hypoplasia, enlarged pulp chambers, and defective dentin. Moreover, taurodontism is commonly observed in the molars (Wright et al., 1994; Wright et al., 1997; Price et al., 1998b; Nieminen et al., 2011). Attrition and dental abscesses are also frequently observed (Crawford and Aldred, 1990). Both the primary and secondary teeth are affected, as they are smaller and spaced (Crawford and Aldred, 1990). Jain et al. also reported the precocious eruption of the permanent molars (Jain et al., 2017).

#### 4.1.3 Dental management

Patients with TDO mainly suffer from dental hypersensitivity, attrition, loss of tooth structure, dental abscesses, esthetic problems, and psychosocial problems (Al-Batayneh, 2012). A comprehensive treatment plan is required to achieve a satisfactory long-term prognosis.

Restorative treatments are required for patients with TDO to recover their tooth shape. Full crowns (prefabricated stainless steel crowns) are beneficial for TDO patients (Fazel et al., 2021), as they can decrease the risk of dental caries and recover the occlusal vertical dimension. For young patients, temporary treatment, such as partial or complete overdentures, may be a potential choice since overdentures can prevent bone loss to prepare the patient for future definitive treatments (Fazel et al., 2021). Meanwhile, patients with TDO are likely to suffer from pulpal disease when the apex is open because teeth with weak enamel and dentin are susceptible to caries and attrition, resulting in pulpal exposure and an early need for endodontic treatment (Fazel et al., 2021). Patients with taurodonts are recommended vital pulp therapy instead of full pulp extirpation (Fazel et al., 2021). Furthermore, careful exploration of additional orifices and canals using magnification can increase the success rate of endodontic treatment (Jafarzadeh et al., 2008).

### 4.2 X-linked hypophosphatemic rickets (XLH)

#### 4.2.1 Etiology

Albright et al. first described familial hypophosphatemic rickets as vitamin D resistant rickets in 1937 (Mitchell and Mitchell, 1957). XLH (OMIM 307800), the most frequent form with a prevalence of 1:20000-60000, is a genetic disease characterized by defective mineralization of bones and tooth dentin, such as osteomalacia and radiolucent dentin (Winters et al., 1958; Opsahl Vital et al., 2012; Salmon et al., 2014; Baroncelli and Mora, 2021). It is caused by mutations in a phosphate-regulating gene with homologies to endopeptidases on the X-chromosome (*PHEX*) on chromosome Xp22.1-22.2 (Gaucher et al., 2009). PHEX regulates FGF23 expression whereas high FGF23 concentration in serum results in hypophosphatemia and low concentration of 1,25-dihydroxyvitamin D by damaging renal reabsorption of phosphate and 1a-hydroxylase activity, as well as increasing the activity of renal 24-hydroxylase (Baroncelli and Mora, 2021). Furthermore, a lack of functional PHEX causes an unnatural increase in the acidic serine- and aspartate-rich motif (ASARM) peptide, which is identified as a PHEX substrate and is derived from matrix extracellular phosphoglycoprotein (MEPE) (Salmon et al., 2013). The accumulation of the MEPE-derived ASARM peptide in XLH dentin results in impaired dentinogenesis. Salmon et al. cultured SHEDs with a phosphorylated ASARM peptide *in vitro* and implanted a phosphorylated ASARM peptide *in vivo*. It has been demonstrated that SHED differentiation and dentin formation are inhibited by the MEPE-derived ASARM peptide, as DSPP expression is decreased while MEPE expression is upregulated (Salmon et al., 2013). It has also been concluded that odontoblast differentiation and dentin mineralization may be impaired by increased MEPE accumulation in the tubules and matrix (Salmon et al., 2014). Therefore, the MEPE-ASARM system is a promising therapeutic target.

#### 4.2.2 Clinical features

##### 4.2.2.1 Systematic features

Patients with XLH suffer from systemic features, including rickets, reduced growth rate, short stature associated with rickets, osteomalacia, and gradual bowing deformities of the lower limbs (Carpenter, 2012; Emma et al., 2019; Haffner et al., 2019). Moreover, daily activities of patients with XLH can be influenced by pain and physical dysfunction (Carpenter et al., 2018).

##### 4.2.2.2 Dental features

Patients with XLH usually have dental defects, including spontaneous periapical abscesses with fistulae that form without a history of trauma or dental caries, prominent pulp horns in the tooth enamel, and enlarged pulp chambers (Baroncelli et al., 2006). In a case reported by Okawa et al., dentin dysplasia of the extracted teeth of patients with XLH, including interglobular dentin, was observed on histopathological examination (Okawa et al., 2022). These observations suggest that dentin dysplasia is a hallmark of dental defects in patients with XLH and may be due to *PHEX* mutation. Enamel dysplasia has also been observed clinically (Souza et al., 2010).

#### 4.2.3 Dental management

Dental treatment of patients with XLH primarily consists of preventive and endodontic management. Dentin dysplasia in the permanent teeth should be considered when formulating dental treatment plans (Okawa et al., 2022). Therefore, preventive treatment is critical for oral care management (Bradley et al., 2021). The dental pulp is likely to be infected by oral bacteria because of defective mineralization of dentin. Therefore, it is important to prevent pulpal infections (Okawa et al., 2022). Maintenance of oral hygiene, pit and fissure sealants, topical fluoride application, and enamel filling are recommended (Souza et al., 2010; Okawa et al., 2022). Patients with XLH should undergo dental examinations at least twice a year (Haffner et al., 2019). Early interventions may prevent serious dental problems (Souza et al., 2010). Endodontic treatment is essential when the pulp is infected. Root canal treatment (RCT) is suitable in most cases (Bradley et al., 2021). Antibiotics are also helpful for treating acute abscesses (Haffner et al., 2019). Moreover, systemic therapy is important for patients with XLH. Classical systemic treatments include phosphorus correction and administration of calcium (Baroncelli and Mora, 2021).

### 4.3 Raine syndrome (RS)

#### 4.3.1 Etiology

RS (OMIM 259775) was first described in 1989 as a syndrome characterized by lethal osteosclerotic bone dysplasia (Raine et al., 1989). It is a rare autosomal recessive (AR) disorder with a prevalence of <1/1,000,000 (Simpson et al., 2007). In 2007, Simpson et al. identified the pathogenic variants in *FAM20C* (NM_020223.3) as the cause of RS (Simpson et al., 2007). In 2009, *FAM20C* variants were reported in children with mild RS phenotype who survived infancy. Therefore, RS can be divided into two types: lethal (LRS) and non-lethal (NLRS) (Simpson et al., 2009). To date, more than 40 variants of *FAM20C* have been identified in patients with LRS or NLRS (Palma-Lara et al., 2021).

FAM20C, a member of FAM20 (Liu et al., 2018a), is implicated in dentinogenesis and odontoblastic differentiation. Dentin defects and reduced levels of odontoblast differentiation markers have been observed in mouse models knock-out of *Fam20c* (Wang et al., 2012). Depletion of *Fam20*c in mouse dental mesenchymal cells leads to reduced expression of *Runx2* and *Osx/Sp7* as well as downregulated transcription of *Dmp1* and *Dspp*, indicating that FAM20C positively regulates odontoblastic differentiation (Liu et al., 2018a). Furthermore, in mouse models with ablation of *Fam20*c, the expression of *Dspp* was reduced in odontoblasts from the root region, and the BMP signaling pathway was inhibited (Li et al., 2022b).

#### 4.3.2 Clinical features

##### 4.3.2.1 Systematic features

LRS features craniofacial, skeletal, and extra skeletal defects including ocular proptosis, midfacial hypoplasia, generalized osteosclerosis with periosteal bone formation, sclerosis of the long bones and skull, and intracerebral calcifications. Most patients with LRS survive for less than 24 h and probably die of respiratory failure (Raine et al., 1989; Palma-Lara et al., 2021). The characteristics of NLRS include mid-facial hypoplasia, a depressed nasal bridge, ocular proptosis, cerebral calcifications, osteosclerosis, microcephaly, and brain calcifications; however, patients with NLRS may vary in clinical features (Palma-Lara et al., 2021).

##### 4.3.2.2 Dental features

Dental abnormalities in patients with RS include enamel and dentin defects (Palma-Lara et al., 2021). Amelogenesis imperfecta, tooth agenesis, periapical and periodontal abscesses, gingival enlargement, palate malformations, and dentinal defects including interglobular dentin and calcospherites have been observed in NLRS patients (Acevedo et al., 2015; Ferreira et al., 2021).

#### 4.3.3 Dental management

LRS patients may die of respiratory failure, therefore a neonatal intensive care team is needed for the respiratory problems. Moreover, multidisciplinary management is essential for supporting growth and achieving better prognosis (Faundes et al., 2014). But few dental treatments have been reported, may be partly due to the fatality of RS.

### 4.4 Hypophostaphasia, HPP

#### 4.4.1 Etiology

Hypophosphatasia (HPP) is a rare systemic genetic disorder resulting from mutations in *ALPL* gene (also known as *TNSALP*), which encodes the tissue-nonspecific isoenzyme of alkaline phosphatase (TNSALP) and leads to diminished ALP activity (Whyte, 2016; Simon et al., 2018). Mutations in the *ALPL* gene can be found in the developing teeth, skeleton, lungs, kidneys, and liver, resulting in dental, skeletal, and extra-skeletal manifestations (Simon et al., 2018). Severe HPP can be explained by AR inheritance, whereas mild HPP can be explained by AD or AR inheritance (Whyte et al., 2015).

*ALPL* gene mutations also lead to defective odontoblastic differentiation, as the canonical Wnt signaling pathway can be impaired by ALPL deficiency. In DPSCs from patients with HPP, there is a decrease in p-GSK3β and active β-catenin, and the expression levels of odontoblastic marker genes, including *DSPP* and *DMP1*, are attenuated. In normal DPSCs, downregulation and upregulation of ALPL inhibited and promoted the levels of p-GSK3β and active β-catenin, respectively. Therefore, the odontoblastic differentiation capacity of DPSCs was impaired (Zhang et al., 2021a).

#### 4.4.2 Clinical features

##### 4.4.2.1 Systematic features

Depending on the complications and patients’ age, HPP is divided into seven major forms: odontohypophosphatasia, adult, childhood (OMIM 241510), infantile (OMIM 241500), perinatal (OMIM 146300), benign prenatal phosphatasia and pseudohypophosphatasia (Whyte, 2016), and the clinical features are variable. Skeletal symptoms include bone and muscle pain, arthralgia, and fractures. Extra skeletal features such as seizures, calcifications in various tissues, and respiratory failure are present in HPP patients (Simon et al., 2018).

##### 4.4.2.2 Dental features

Dental complications can be present in mild forms, such as childhood HPP, adult HPP and odontohypophosphatasia (Reibel et al., 2009). Children with HPP usually suffer from the premature loss of deciduous teeth resulting from dentin, cementum, alveolar bone dysplasia, or aplasia (Zhang et al., 2021a). Moreover, permanent dentition can also be affected, as large pulp chambers can be observed in the crown, dentin resorption, and impaired dentin mineralization (Olsson et al., 1996). Delayed dentin formation and enamel defects have also been reported (Reibel et al., 2009).

#### 4.4.3 Dental management

In 2015, enzyme replacement therapy (asfotase alfa) was approved as a valid treatment for HPP patients (Whyte, 2016). Dental care is of great importance in management plans. Patients with HPP usually experience early exfoliation of many teeth; therefore, dentures can be helpful for recovering speech and mastication (Whyte, 2016). Furthermore, Zhang et al. suggested that systemic LiCl injections can be a promising therapy for patients with HPP, as LiCl can improve dentin mineralization, dentin mineral density, and the height and bone mass of alveolar bone in mouse models with ALPL depletion. Mechanistically, LiCl activates the canonical Wnt pathway, enhancing the differentiation of HPP DPSCs into odontoblasts (Zhang et al., 2021a).

### 4.5 Other genetic syndromes with dentin defects

Apart from the abovementioned syndromes, there are still systemic diseases with dentin defects (Su et al., 2023). Schimke immuno-osseous dysplasia (SIOD, OMIM 242900) and Elsahy-Waters syndrome (EWS, OMIM 211380) are discussed in this section, because their gene mutations may impair odontoblastic differentiation of DMSCs (Su et al., 2023). Although the pathological mechanism by which the gene mutations affect odontoblastic differentiation has not yet been revealed, how they influence osteoblasts or lead to skeletal deformations still poses a possibility for future research.

SIOD is a rare AR genetic syndrome resulting from bi-allelic mutations in *SMARCAL1* (SWI/SNF-related, matrix-associated, actin-dependent regulator of chromatin, subfamily a-like 1) (Boerkoel et al., 2002). *SMARCAL1* encodes a protein from the sucrose non-fermenting 2 (SNF2) family, which serves as a DNA annealing helicase involved in chromatin remodeling (Yusufzai and Kadonaga, 2008), and SMARCAL1 is massively present in developing human teeth (Morimoto et al., 2012). In cultured SIOD fibroblasts, Wnt3a, BMP4, and TGF-β1 signaling is altered, which may shed light on the possible mechanism of SIOD dental anomalies (Morimoto et al., 2012). It is characterized by spondyloepiphyseal dysplasia, T cell immunodeficiency, renal dysfunction, facial dysmorphism, and dental anomalies (Schimke et al., 1971; Spranger et al., 1991; Boerkoel et al., 2000; Morimoto et al., 2012). Morimoto et al. reported that 66% of patients with SIOD and biallelic *SMARCAL1* mutations had microdontia, hypodontia, or malformed molars (Morimoto et al., 2012). In a case report by da Fonseca, the panoramic film showed that both the primary and permanent teeth had bulbous crowns with marked cervical constriction, the pulp chambers were smaller or obliterated, and the roots were thinner, similar to the dental features of dentinogenesis imperfecta (DI) type II (da Fonseca, 2000). These characteristic dental anomalies facilitate the diagnosis of SIOD (Gendronneau et al., 2014).

EWS, also known as brachioskeletogenital syndrome (BSGS), is an ultrarare AR syndrome caused by mutations in cadherin-11 (*CDH11*) (Taskiran et al., 2017; Castori et al., 2018). CDH11 mediates cell-cell adhesion and migration; moreover, it is critical for mesenchymal stem cell differentiation (Alimperti and Andreadis, 2015). In contrast, loss-of-function CDH11 delays osteogenic differentiation, which may cause craniofacial defects in EWS patients (Taskiran et al., 2017). EWS is characterized by skeletal and craniofacial malformations, intelligence disorders, and dental anomalies. For example, patients with EWS may present with brachycephaly, an underdeveloped maxilla, mandibular prognathism, midface hypoplasia, hypertelorism, proptosis, cervical vertebral fusion, and hypogenitalism (el-Sahy and Waters, 1971). Dental anomalies include radicular dentin dysplasia with consequent obliterated pulp chambers, apical translucent “cysts”, recurrent infections, and early loss of teeth (Castori et al., 2010). In conclusion, the OMIM, gene mutations, and dental features of these genetic syndromes mentioned above are clearly listed in Table 2.

**TABLE 2:** Genetic syndromes with dentin defects caused by impaired odontoblast differentiation.

Diseases	OMIM	Genes	Dental features
Tricho-dento-osseous (TDO) syndrome	190320	*DLX3*	Thin enamel, thin dentin, taurodontism, attritions, dental abscesses, precocious eruption of permanent molars
X-linked hypophosphatemic rickets (XLH)	307800	*PHEX*	Spontaneous periapical abscesses with fistulae that form without trauma history or dental caries, prominent pulp horns into the tooth enamel, enlarged pulp chambers, enamel dysplasia
Raine syndrome	259775	*FAM20C*	Amelogenesis imperfecta, tooth agenesis, periapical and periodontal abscesses, gingival enlargement, palate malformations, and dentinal defects, including interglobular dentin and calcospherites
Hypophosphatasia (HPP)	—	*ALPL*	Premature loss of the deciduous teeth, large pulp chambers, dentin resorption, impaired dentin mineralization, delayed dentine formation, enamel defects
Schimke immune-osseous dysplasia (SIOD)	242900	*SMARCAL1*	Microdontia, hypodontia, or malformed molars, dentinogenesis imperfecta (DI) type II
Elsahy-Waters syndrome (EWS)	211380	*CDH11*	Radicular dentin dysplasia with consequent obliterated pulp chambers, apical translucent “cysts”, recurrent infections, and early loss of teeth

## 5 Conclusion

Since the discovery of DPSCs in 2000 (Gronthos et al., 2000), the past two decades have witnessed the development of research on DMSCs (Sui et al., 2020). The odontoblastic differentiation of DMSCs is a critical step during dentin formation and is regulated by signaling pathways, TFs, and posttranscriptional and epigenetic regulation at the molecular level. In this review, recent achievements are summarized, and an atlas of the regulatory mechanisms provides a deep understanding of the odontoblastic differentiation of DMSCs. In addition, this review provides an overview of the etiology, clinical features, and dental management of genetic syndromes associated with dentin defects caused by impaired odontoblast differentiation, including TDO syndrome, XLH, RS, HPP, SIOD, and EWS. Therefore, a comprehensive mechanistic insight into the odontoblastic differentiation of DMSCs could shed light on the molecular mechanisms of known and unknown genetic syndromes and identify promising treatment targets for dentin defects.

## References

[B1] Acevedo A. C. Poulter J. A. Alves P. G. De Lima C. L. Castro L. C. Yamaguti P. M. (2015). Variability of systemic and oro-dental phenotype in two families with non-lethal Raine syndrome with FAM20C mutations. BMC Med. Genet. 16, 8. 10.1186/s12881-015-0154-5 25928877 PMC4422040

[B2] Al-Batayneh O. B. (2012). Tricho-dento-osseous syndrome: diagnosis and dental management. Int. J. Dent. 2012, 514692. 10.1155/2012/514692 22969805 PMC3434396

[B3] Alimperti S. Andreadis S. T. (2015). CDH2 and CDH11 act as regulators of stem cell fate decisions. Stem Cell Res. 14, 270–282. 10.1016/j.scr.2015.02.002 25771201 PMC4439315

[B4] Ambros V. (2004). The functions of animal microRNAs. Nature 431, 350–355. 10.1038/nature02871 15372042

[B5] Armiñán A. Gandía C. Bartual M. García-Verdugo J. M. Lledó E. Mirabet V. (2009). Cardiac differentiation is driven by NKX2.5 and GATA4 nuclear translocation in tissue-specific mesenchymal stem cells. Stem Cells Dev. 18, 907–918. 10.1089/scd.2008.0292 18983250

[B6] Baroncelli G. I. Mora S. (2021). X-linked hypophosphatemic rickets: multisystemic disorder in children requiring multidisciplinary management. Front. Endocrinol. (Lausanne) 12, 688309. 10.3389/fendo.2021.688309 34421819 PMC8378329

[B7] Bae C. H. Kim T. H. Ko S. O. Lee J. C. Yang X. Cho E. S. (2015). Wntless regulates dentin apposition and root elongation in the mandibular molar. J. Dent. Res. 94, 439–445. 10.1177/0022034514567198 25595365 PMC4814015

[B8] Bae J. M. Clarke J. C. Rashid H. Adhami M. D. Mccullough K. Scott J. S. (2018). Specificity protein 7 is required for proliferation and differentiation of ameloblasts and odontoblasts. J. Bone Min. Res. 33, 1126–1140. 10.1002/jbmr.3401 PMC6002875 29405385

[B9] Bai Y. Cheng X. Liu X. Guo Q. Wang Z. Fu Y. (2023). Transforming growth factor-β1 promotes early odontoblastic differentiation of dental pulp stem cells via activating AKT, Erk1/2 and p38 MAPK pathways. J. Dent. Sci. 18, 87–94. 10.1016/j.jds.2022.06.027 36643229 PMC9831829

[B10] Bai Y. Liu X. Li J. Wang Z. Guo Q. Xiao M. (2022). Stage-dependent regulation of dental pulp stem cell odontogenic differentiation by transforming growth factor-β1. Stem Cells Int. 2022, 2361376. 10.1155/2022/2361376 36338026 PMC9629931

[B11] Baron R. Kneissel M. (2013). WNT signaling in bone homeostasis and disease: from human mutations to treatments. Nat. Med. 19, 179–192. 10.1038/nm.3074 23389618

[B12] Baroncelli G. I. Angiolini M. Ninni E. Galli V. Saggese R. Giuca M. R. (2006). Prevalence and pathogenesis of dental and periodontal lesions in children with X-linked hypophosphatemic rickets. Eur. J. Paediatr. Dent. 7, 61–66. 16842025

[B13] Boerkoel C. F. O'Neill S. André J. L. Benke P. J. Bogdanovíć R. Bulla M. (2000). Manifestations and treatment of Schimke immuno-osseous dysplasia: 14 new cases and a review of the literature. Eur. J. Pediatr. 159, 1–7. 10.1007/s004310050001 10653321

[B14] Boerkoel C. F. Takashima H. John J. Yan J. Stankiewicz P. Rosenbarker L. (2002). Mutant chromatin remodeling protein SMARCAL1 causes Schimke immuno-osseous dysplasia. Nat. Genet. 30, 215–220. 10.1038/ng821 11799392

[B15] Bradley H. Dutta A. Philpott R. (2021). Presentation and non-surgical endodontic treatment of two patients with X-linked hypophosphatemia: a case report. Int. Endod. J. 54, 1403–1414. 10.1111/iej.13520 33749833

[B16] Busby T. Chen Y. Godfrey T. C. Rehan M. Wildman B. J. Smith C. M. (2021). Baf45a mediated chromatin remodeling promotes transcriptional activation for osteogenesis and odontogenesis. Front. Endocrinol. (Lausanne) 12, 763392. 10.3389/fendo.2021.763392 35046892 PMC8762305

[B17] Cai X. Gong P. Huang Y. Lin Y. (2011). Notch signalling pathway in tooth development and adult dental cells. Cell Prolif. 44, 495–507. 10.1111/j.1365-2184.2011.00780.x 21973022 PMC6495681

[B18] Camilleri S. Mcdonald F. (2006). Runx2 and dental development. Eur. J. Oral Sci. 114, 361–373. 10.1111/j.1600-0722.2006.00399.x 17026500

[B19] Carinci F. Papaccio G. Laino G. Palmieri A. Brunelli G. D'Aquino R. (2008). Comparison between genetic portraits of osteoblasts derived from primary cultures and osteoblasts obtained from human pulpar stem cells. J. Craniofac Surg. 19, 616–625. 10.1097/SCS.0b013e31816aabc8 18520373

[B20] Carpenter T. O. (2012). The expanding family of hypophosphatemic syndromes. J. Bone Min. Metab. 30, 1–9. 10.1007/s00774-011-0340-2 22167381

[B21] Carpenter T. O. Whyte M. P. Imel E. A. Boot A. M. Högler W. Linglart A. (2018). Burosumab therapy in children with X-linked hypophosphatemia. N. Engl. J. Med. 378, 1987–1998. 10.1056/NEJMoa1714641 29791829

[B22] Castori M. Cascone P. Valiante M. Laino L. Iannetti G. Hennekam R. C. (2010). Elsahy-waters syndrome: evidence for autosomal recessive inheritance. Am. J. Med. Genet. A 152a, 2810–2815. 10.1002/ajmg.a.33634 20949527

[B23] Castori M. Ott C. E. Bisceglia L. Leone M. P. Mazza T. Castellana S. (2018). A novel mutation in CDH11, encoding cadherin-11, cause Branchioskeletogenital (Elsahy-Waters) syndrome. Am. J. Med. Genet. A 176, 2028–2033. 10.1002/ajmg.a.40379 30194892

[B24] Chen G. Deng C. Li Y. P. (2012). TGF-β and BMP signaling in osteoblast differentiation and bone formation. Int. J. Biol. Sci. 8, 272–288. 10.7150/ijbs.2929 22298955 PMC3269610

[B25] Chen J. Lan Y. Baek J. A. Gao Y. Jiang R. (2009a). Wnt/beta-catenin signaling plays an essential role in activation of odontogenic mesenchyme during early tooth development. Dev. Biol. 334, 174–185. 10.1016/j.ydbio.2009.07.015 19631205 PMC2752344

[B26] Chen L. Song Z. Huang S. Wang R. Qin W. Guo J. (2016a). lncRNA DANCR suppresses odontoblast-like differentiation of human dental pulp cells by inhibiting wnt/β-catenin pathway. Cell Tissue Res. 364, 309–318. 10.1007/s00441-015-2333-2 26646542

[B27] Chen L. Song Z. Wu J. Huang Q. Shen Z. Wei X. (2020). LncRNA DANCR sponges miR-216a to inhibit odontoblast differentiation through upregulating c-Cbl. Exp. Cell Res. 387, 111751. 10.1016/j.yexcr.2019.111751 31805275

[B28] Chen L. Zheng L. Jiang J. Gui J. Zhang L. Huang Y. (2016b). Calcium hydroxide-induced proliferation, migration, osteogenic differentiation, and mineralization via the mitogen-activated protein kinase pathway in human dental pulp stem cells. J. Endod. 42, 1355–1361. 10.1016/j.joen.2016.04.025 27395474

[B29] Chen S. Gluhak-Heinrich J. Martinez M. Li T. Wu Y. Chuang H. H. (2008). Bone morphogenetic protein 2 mediates dentin sialophosphoprotein expression and odontoblast differentiation via NF-Y signaling. J. Biol. Chem. 283, 19359–19370. 10.1074/jbc.M709492200 18424784 PMC2443643

[B30] Chen T. Dent S. Y. (2014). Chromatin modifiers and remodellers: regulators of cellular differentiation. Nat. Rev. Genet. 15, 93–106. 10.1038/nrg3607 24366184 PMC3999985

[B31] Chen Z. Couble M. L. Mouterfi N. Magloire H. Chen Z. Bleicher F. (2009b). Spatial and temporal expression of KLF4 and KLF5 during murine tooth development. Arch. Oral Biol. 54, 403–411. 10.1016/j.archoralbio.2009.02.003 19268913

[B32] Chen Z. Li W. Wang H. Wan C. Luo D. Deng S. (2016c). Klf10 regulates odontoblast differentiation and mineralization via promoting expression of dentin matrix protein 1 and dentin sialophosphoprotein genes. Cell Tissue Res. 363, 385–398. 10.1007/s00441-015-2260-2 26310138 PMC5006385

[B33] Chen Z. Xie H. Yuan J. Lan Y. Xie Z. (2021). Krüppel-like factor 6 promotes odontoblastic differentiation through regulating the expression of dentine sialophosphoprotein and dentine matrix protein 1 genes. Int. Endod. J. 54, 572–584. 10.1111/iej.13447 33200415

[B34] Chen Z. Zhang Q. Wang H. Li W. Wang F. Wan C. (2017). Klf5 mediates odontoblastic differentiation through regulating dentin-specific extracellular matrix gene expression during mouse tooth development. Sci. Rep. 7, 46746. 10.1038/srep46746 28440310 PMC5404268

[B35] Choi S. J. Song I. S. Feng J. Q. Gao T. Haruyama N. Gautam P. (2010). Mutant DLX 3 disrupts odontoblast polarization and dentin formation. Dev. Biol. 344, 682–692. 10.1016/j.ydbio.2010.05.499 20510228 PMC2945701

[B36] Clevers H. Nusse R. (2012). Wnt/β-catenin signaling and disease. Cell 149, 1192–1205. 10.1016/j.cell.2012.05.012 22682243

[B37] Cobourne M. T. Sharpe P. T. (2003). Tooth and jaw: molecular mechanisms of patterning in the first branchial arch. Arch. Oral Biol. 48, 1–14. 10.1016/s0003-9969(02)00208-x 12615136

[B38] Coulombe P. Meloche S. (2007). Atypical mitogen-activated protein kinases: structure, regulation and functions. Biochim. Biophys. Acta 1773, 1376–1387. 10.1016/j.bbamcr.2006.11.001 17161475

[B39] Crawford P. J. Aldred M. J. (1990). Amelogenesis imperfecta with taurodontism and the tricho-dento-osseous syndrome: separate conditions or a spectrum of disease? Clin. Genet. 38, 44–50. 10.1111/j.1399-0004.1990.tb03546.x 2387085

[B40] Cruciat C. M. Niehrs C. (2013). Secreted and transmembrane wnt inhibitors and activators. Cold Spring Harb. Perspect. Biol. 5, a015081. 10.1101/cshperspect.a015081 23085770 PMC3578365

[B41] Cui D. Xiao J. Zhou Y. Zhou X. Liu Y. Peng Y. (2019). Epiregulin enhances odontoblastic differentiation of dental pulp stem cells via activating MAPK signalling pathway. Cell Prolif. 52, e12680. 10.1111/cpr.12680 31454111 PMC6869433

[B42] D'Aquino R. Graziano A. Sampaolesi M. Laino G. Pirozzi G. De Rosa A. (2007). Human postnatal dental pulp cells co-differentiate into osteoblasts and endotheliocytes: a pivotal synergy leading to adult bone tissue formation. Cell Death Differ. 14, 1162–1171. 10.1038/sj.cdd.4402121 17347663

[B43] D'Souza R. N. Aberg T. Gaikwad J. Cavender A. Owen M. Karsenty G. (1999). Cbfa1 is required for epithelial-mesenchymal interactions regulating tooth development in mice. Development 126, 2911–2920. 10.1242/dev.126.13.2911 10357935

[B44] Da Fonseca M. A. (2000). Dental findings in the Schimke immuno-osseous dysplasia. Am. J. Med. Genet. 93, 158–160. 10.1002/1096-8628(20000717)93:2<158::aid-ajmg14>3.0.co;2-4 10869120

[B45] Du J. Ma Y. Ma P. Wang S. Fan Z. (2013). Demethylation of epiregulin gene by histone demethylase FBXL11 and BCL6 corepressor inhibits osteo/dentinogenic differentiation. Stem Cells 31, 126–136. 10.1002/stem.1255 23074094

[B46] Duan P. Bonewald L. F. (2016). The role of the wnt/β-catenin signaling pathway in formation and maintenance of bone and teeth. Int. J. Biochem. Cell Biol. 77, 23–29. 10.1016/j.biocel.2016.05.015 27210503 PMC4958569

[B47] Duncan H. F. Kobayashi Y. Yamauchi Y. Quispe-Salcedo A. Chao Feng Z. Huang J. (2022). The critical role of MMP13 in regulating tooth development and reactionary dentinogenesis repair through the wnt signaling pathway. Front. Cell Dev. Biol. 10, 883266. 10.3389/fcell.2022.883266 35531096 PMC9068941

[B48] Duverger O. Zah A. Isaac J. Sun H. W. Bartels A. K. Lian J. B. (2012). Neural crest deletion of Dlx3 leads to major dentin defects through down-regulation of Dspp. J. Biol. Chem. 287, 12230–12240. 10.1074/jbc.M111.326900 22351765 PMC3320974

[B49] Eiraku M. Hirata Y. Takeshima H. Hirano T. Kengaku M. (2002). Delta/notch-like epidermal growth factor (EGF)-related receptor, a novel EGF-like repeat-containing protein targeted to dendrites of developing and adult central nervous system neurons. J. Biol. Chem. 277, 25400–25407. 10.1074/jbc.M110793200 11950833

[B50] El-Sahy N. I. Waters W. R. (1971). The branchio-skeleto-genital syndrome. A new hereditary syndrome. Plast. Reconstr. Surg. 48, 542–550. 10.1097/00006534-197112000-00004 5141271

[B51] Emma F. Cappa M. Antoniazzi F. Bianchi M. L. Chiodini I. Eller Vainicher C. (2019). X-Linked hypophosphatemic rickets: an Italian experts' opinion survey. Ital. J. Pediatr. 45, 67. 10.1186/s13052-019-0654-6 31151476 PMC6545008

[B52] Fang F. Zhang K. Chen Z. Wu B. (2019). Noncoding RNAs: new insights into the odontogenic differentiation of dental tissue-derived mesenchymal stem cells. Stem Cell Res. Ther. 10, 297. 10.1186/s13287-019-1411-x 31547871 PMC6757432

[B53] Faundes V. Castillo-Taucher S. Gonzalez-Hormazabal P. Chandler K. Crosby A. Chioza B. (2014). Raine syndrome: an overview. Eur. J. Med. Genet. 57, 536–542. 10.1016/j.ejmg.2014.07.001 25019372

[B54] Fazel M. Afshari E. Jarrahi N. (2021). Dental management of tricho-dento-osseous syndrome in adolescent patients: literature review and case presentation. Dent. Res. J. (Isfahan) 18, 98. 10.4103/1735-3327.330879 35003563 PMC8672132

[B55] Ferreira L. D. Leal G. F. De Oliveira J. R. M. (2021). Non-lethal raine syndrome report lacking characteristic clinical features. J. Mol. Neurosci. 71, 2482–2486. 10.1007/s12031-021-01873-z 34259997

[B56] Fu T. Liu Y. Huang X. Guo Y. Shen J. Shen H. (2022). lncRNA SNHG1 regulates odontogenic differentiation of human dental pulp stem cells via miR-328-3p/Wnt/β-catenin pathway. Stem Cell Res. Ther. 13, 311. 10.1186/s13287-022-02979-w 35841022 PMC9284872

[B57] Gan L. Liu Y. Cui D. Pan Y. Zheng L. Wan M. (2020). Dental tissue-derived human mesenchymal stem cells and their potential in therapeutic application. Stem Cells Int. 2020, 8864572. 10.1155/2020/8864572 32952572 PMC7482010

[B58] Gao R. Dong R. Du J. Ma P. Wang S. Fan Z. (2013). Depletion of histone demethylase KDM2A inhibited cell proliferation of stem cells from apical papilla by de-repression of p15INK4B and p27Kip1. Mol. Cell Biochem. 379, 115–122. 10.1007/s11010-013-1633-7 23559091

[B59] Gaucher C. Walrant-Debray O. Nguyen T. M. Esterle L. Garabédian M. Jehan F. (2009). PHEX analysis in 118 pedigrees reveals new genetic clues in hypophosphatemic rickets. Hum. Genet. 125, 401–411. 10.1007/s00439-009-0631-z 19219621

[B60] Gendronneau M. Kérourédan O. Taque S. Sixou J. L. Bonnaure-Mallet M. (2014). Dental abnormalities and preventive oral care in Schimke immuno-osseous dysplasia. Eur. Arch. Paediatr. Dent. 15, 217–221. 10.1007/s40368-013-0099-3 24327104 PMC5127816

[B61] Ghoul-Mazgar S. Hotton D. Lézot F. Blin-Wakkach C. Asselin A. Sautier J. M. (2005). Expression pattern of Dlx3 during cell differentiation in mineralized tissues. Bone 37, 799–809. 10.1016/j.bone.2005.03.020 16172034

[B62] Gilboa L. Wells R. G. Lodish H. F. Henis Y. I. (1998). Oligomeric structure of type I and type II transforming growth factor beta receptors: homodimers form in the ER and persist at the plasma membrane. J. Cell Biol. 140, 767–777. 10.1083/jcb.140.4.767 9472030 PMC2141740

[B63] Gong Y. Yuan S. Sun J. Wang Y. Liu S. Guo R. (2020). R-spondin 2 induces odontogenic differentiation of dental pulp stem/progenitor cells via regulation of wnt/β-catenin signaling. Front. Physiol. 11, 918. 10.3389/fphys.2020.00918 32848860 PMC7426510

[B64] Gronostajski R. M. (2000). Roles of the NFI/CTF gene family in transcription and development. Gene 249, 31–45. 10.1016/s0378-1119(00)00140-2 10831836

[B65] Gronthos S. Mankani M. Brahim J. Robey P. G. Shi S. (2000). Postnatal human dental pulp stem cells (DPSCs) *in vitro* and *in vivo* . Proc. Natl. Acad. Sci. U. S. A. 97, 13625–13630. 10.1073/pnas.240309797 11087820 PMC17626

[B66] Gu D. Liu H. Qiu X. Yu Y. Tang X. Liu C. (2022). Erythropoietin induces odontoblastic differentiation of human-derived pulp stem cells via EphB4-Mediated MAPK signaling pathway. Oral Dis. 2022, 14486. 10.1111/odi.14486 36577689

[B67] Haffner D. Emma F. Eastwood D. M. Duplan M. B. Bacchetta J. Schnabel D. (2019). Clinical practice recommendations for the diagnosis and management of X-linked hypophosphataemia. Nat. Rev. Nephrol. 15, 435–455. 10.1038/s41581-019-0152-5 31068690 PMC7136170

[B68] Han N. Zheng Y. Li R. Li X. Zhou M. Niu Y. (2014). β-catenin enhances odontoblastic differentiation of dental pulp cells through activation of Runx2. PLoS One 9, e88890. 10.1371/journal.pone.0088890 24520423 PMC3919828

[B69] Hao H.-X. Xie Y. Zhang Y. Charlat O. Oster E. Avello M. (2012). ZNRF3 promotes Wnt receptor turnover in an R-spondin-sensitive manner. Nature 485, 195–200. 10.1038/nature11019 22575959

[B70] Hassan T. Qiu Y. Hasan M. R. Saito T. (2022). Effects of dentin phosphophoryn-derived RGD peptides on the differentiation and mineralization of human dental pulp stem cells *in vitro* . Biomedicines 10, 2781. 10.3390/biomedicines10112781 36359301 PMC9687143

[B71] Hata A. Chen Y. G. (2016). TGF-Β signaling from receptors to Smads. Cold Spring Harb. Perspect. Biol. 8, a022061. 10.1101/cshperspect.a022061 27449815 PMC5008074

[B72] He F. Tan Y. Yang Z. (2003). The roles of Notch2-Delta signaling in the differentiation of long-term cultured human dental pulp cells. Hua Xi Kou Qiang Yi Xue Za Zhi 21, 344–371. 14650983

[B73] He P. Zheng L. Zhou X. (2022). IGFs in dentin formation and regeneration: progress and remaining challenges. Stem Cells Int. 2022, 3737346. 10.1155/2022/3737346 35432548 PMC9007658

[B74] He W. X. Niu Z. Y. Zhao S. L. Jin W. L. Gao J. Smith A. J. (2004). TGF-beta activated Smad signalling leads to a Smad3-mediated down-regulation of DSPP in an odontoblast cell line. Arch. Oral Biol. 49, 911–918. 10.1016/j.archoralbio.2004.05.005 15353247

[B75] He W. Zhang J. Niu Z. Yu Q. Wang Z. Zhang R. (2014). Regulatory interplay between NFIC and TGF-β1 in apical papilla-derived stem cells. J. Dent. Res. 93, 496–501. 10.1177/0022034514525200 24570148

[B76] Henis Y. I. Moustakas A. Lin H. Y. Lodish H. F. (1994). The types II and III transforming growth factor-beta receptors form homo-oligomers. J. Cell Biol. 126, 139–154. 10.1083/jcb.126.1.139 8027173 PMC2120107

[B77] Ho L. Crabtree G. R. (2010). Chromatin remodelling during development. Nature 463, 474–484. 10.1038/nature08911 20110991 PMC3060774

[B78] Hoodless P. A. Haerry T. Abdollah S. Stapleton M. O'Connor M. B. Attisano L. (1996). MADR1, a MAD-related protein that functions in BMP2 signaling pathways. Cell 85, 489–500. 10.1016/s0092-8674(00)81250-7 8653785

[B79] Hosoya A. Yukita A. Ninomiya T. Hiraga T. Yoshiba K. Yoshiba N. (2013). Localization of SUMOylation factors and Osterix in odontoblast lineage cells during dentin formation and regeneration. Histochem Cell Biol. 140, 201–211. 10.1007/s00418-013-1076-y 23354182

[B80] Huang G. T. Sonoyama W. Liu Y. Liu H. Wang S. Shi S. (2008). The hidden treasure in apical papilla: the potential role in pulp/dentin regeneration and bioroot engineering. J. Endod. 34, 645–651. 10.1016/j.joen.2008.03.001 18498881 PMC2653220

[B81] Huang X. Liu F. Hou J. Chen K. (2019). Inflammation-induced overexpression of microRNA-223-3p regulates odontoblastic differentiation of human dental pulp stem cells by targeting SMAD3. Int. Endod. J. 52, 491–503. 10.1111/iej.13032 30368846

[B82] Ikeda E. Yagi K. Kojima M. Yagyuu T. Ohshima A. Sobajima S. (2008). Multipotent cells from the human third molar: feasibility of cell-based therapy for liver disease. Differentiation 76, 495–505. 10.1111/j.1432-0436.2007.00245.x 18093227

[B83] Ishkitiev N. Yaegaki K. Imai T. Tanaka T. Nakahara T. Ishikawa H. (2012). High-purity hepatic lineage differentiated from dental pulp stem cells in serum-free medium. J. Endod. 38, 475–480. 10.1016/j.joen.2011.12.011 22414832

[B84] Islam M. Lurie A. G. Reichenberger E. (2005). Clinical features of tricho-dento-osseous syndrome and presentation of three new cases: an addition to clinical heterogeneity. Oral Surg. Oral Med. Oral Pathol. Oral Radiol. Endod. 100, 736–742. 10.1016/j.tripleo.2005.04.017 16301156

[B85] Iwamoto T. Nakamura T. Ishikawa M. Yoshizaki K. Sugimoto A. Ida-Yonemochi H. (2017). Pannexin 3 regulates proliferation and differentiation of odontoblasts via its hemichannel activities. PLoS One 12, e0177557. 10.1371/journal.pone.0177557 28494020 PMC5426780

[B86] Jafarzadeh H. Azarpazhooh A. Mayhall J. T. (2008). Taurodontism: a review of the condition and endodontic treatment challenges. Int. Endod. J. 41, 375–388. 10.1111/j.1365-2591.2008.01388.x 18363703

[B87] Jain P. Kaul R. Saha S. Sarkar S. (2017). Tricho-dento-osseous syndrome and precocious eruption. J. Clin. Exp. Dent. 9, e494–e497. 10.4317/jced.53348 28298997 PMC5347304

[B88] Janda C. Y. Dang L. T. You C. Chang J. De Lau W. Zhong Z. A. (2017). Surrogate Wnt agonists that phenocopy canonical Wnt and β-catenin signalling. Nature 545, 234–237. 10.1038/nature22306 28467818 PMC5815871

[B89] Jang H. O. Ahn T. Y. Ju J. M. Bae S. K. Kim H. R. Kim D. S. (2022). Odontogenic differentiation-induced tooth regeneration by Psoralea corylifolia L. Curr. Issues Mol. Biol. 44, 2300–2308. 10.3390/cimb44050156 35678685 PMC9164060

[B90] Jiang L. Ayre W. N. Melling G. E. Song B. Wei X. Sloan A. J. (2020). Liposomes loaded with transforming growth factor β1 promote odontogenic differentiation of dental pulp stem cells. J. Dent. 103, 103501. 10.1016/j.jdent.2020.103501 33068710

[B91] Jin H. Park J. Y. Choi H. Choung P. H. (2013). HDAC inhibitor trichostatin A promotes proliferation and odontoblast differentiation of human dental pulp stem cells. Tissue Eng. Part A 19, 613–624. 10.1089/ten.TEA.2012.0163 23013422

[B92] Johnson G. L. Lapadat R. (2002). Mitogen-activated protein kinase pathways mediated by ERK, JNK, and p38 protein kinases. Science 298, 1911–1912. 10.1126/science.1072682 12471242

[B93] Kakugawa S. Langton P. F. Zebisch M. Howell S. A. Chang T.-H. Liu Y. (2015). Notum deacylates Wnt proteins to suppress signalling activity. Nature 519, 187–192. 10.1038/nature14259 25731175 PMC4376489

[B94] Kamiunten T. Ideno H. Shimada A. Nakamura Y. Kimura H. Nakashima K. (2015). Coordinated expression of H3K9 histone methyltransferases during tooth development in mice. Histochem Cell Biol. 143, 259–266. 10.1007/s00418-014-1284-0 25294562

[B95] Karanxha L. Park S. J. Son W. J. Nör J. E. Min K. S. (2013). Combined effects of simvastatin and enamel matrix derivative on odontoblastic differentiation of human dental pulp cells. J. Endod. 39, 76–82. 10.1016/j.joen.2012.10.013 23228261 PMC3812675

[B96] Khalid M. Hodjat M. Baeeri M. Rahimifard M. Bayrami Z. Abdollahi M. (2022). Lead inhibits the odontogenic differentiation potential of dental pulp stem cells by affecting WNT1/β-catenin signaling and related miRNAs expression. Toxicol Vitro 83, 105422. 10.1016/j.tiv.2022.105422 35738543

[B97] Kim J. W. Choi H. Jeong B. C. Oh S. H. Hur S. W. Lee B. N. (2014). Transcriptional factor ATF6 is involved in odontoblastic differentiation. J. Dent. Res. 93, 483–489. 10.1177/0022034514525199 24570149 PMC6728569

[B98] Kim J. Y. Kim D. S. Auh Q. S. Yi J. K. Moon S. U. Kim E. C. (2017). Role of protein phosphatase 1 in angiogenesis and odontoblastic differentiation of human dental pulp cells. J. Endod. 43, 417–424. 10.1016/j.joen.2016.10.013 28231980

[B99] Kimura M. Saito A. Onodera S. Nakamura T. Suematsu M. Shintani S. (2022). The concurrent stimulation of Wnt and FGF8 signaling induce differentiation of dental mesenchymal cells into odontoblast-like cells. Med. Mol. Morphol. 55, 8–19. 10.1007/s00795-021-00297-3 34739612 PMC8885561

[B100] Ko C. S. Chen J. H. Su W. T. (2020). Stem cells from human exfoliated deciduous teeth: a concise review. Curr. Stem Cell Res. Ther. 15, 61–76. 10.2174/1574888X14666191018122109 31648649

[B101] Komori T. Yagi H. Nomura S. Yamaguchi A. Sasaki K. Deguchi K. (1997). Targeted disruption of Cbfa1 results in a complete lack of bone formation owing to maturational arrest of osteoblasts. Cell 89, 755–764. 10.1016/s0092-8674(00)80258-5 9182763

[B102] Koo B.-K. Spit M. Jordens I. Low T. Y. Stange D. E. Van De Wetering M. (2012). Tumour suppressor RNF43 is a stem-cell E3 ligase that induces endocytosis of Wnt receptors. Nature 488, 665–669. 10.1038/nature11308 22895187

[B103] Kopp F. Mendell J. T. (2018). Functional classification and experimental dissection of long noncoding RNAs. Cell 172, 393–407. 10.1016/j.cell.2018.01.011 29373828 PMC5978744

[B104] Kretz M. Webster D. E. Flockhart R. J. Lee C. S. Zehnder A. Lopez-Pajares V. (2012). Suppression of progenitor differentiation requires the long noncoding RNA ANCR. Genes Dev. 26, 338–343. 10.1101/gad.182121.111 22302877 PMC3289881

[B105] Lee E. C. Kim Y. M. Lim H. M. Ki G. E. Seo Y. K. (2020). The histone deacetylase inhibitor (MS-275) promotes differentiation of human dental pulp stem cells into odontoblast-like cells independent of the MAPK signaling system. Int. J. Mol. Sci. 21, 5771. 10.3390/ijms21165771 32796747 PMC7460873

[B106] Lee H. K. Lee D. S. Park S. J. Cho K. H. Bae H. S. Park J. C. (2014). Nuclear factor I-C (NFIC) regulates dentin sialophosphoprotein (DSPP) and E-cadherin via control of Krüppel-like factor 4 (KLF4) during dentinogenesis. J. Biol. Chem. 289, 28225–28236. 10.1074/jbc.M114.568691 25138274 PMC4192478

[B107] Lee S. I. Kim S. Y. Park K. R. Kim E. C. (2016). Baicalein promotes angiogenesis and odontoblastic differentiation via the BMP and wnt pathways in human dental pulp cells. Am. J. Chin. Med. 44, 1457–1472. 10.1142/S0192415X16500816 27776430

[B108] Lesot H. Lisi S. Peterkova R. Peterka M. Mitolo V. Ruch J. V. (2001). Epigenetic signals during odontoblast differentiation. Adv. Dent. Res. 15, 8–13. 10.1177/08959374010150012001 12640731

[B109] Li C. Xie X. Liu Z. Yang J. Zuo D. Xu S. (2021). Neu5Ac induces human dental pulp stem cell osteo-/odontoblastic differentiation by enhancing MAPK/ERK pathway activation. Stem Cells Int. 2021, 5560872. 10.1155/2021/5560872 34603453 PMC8483915

[B110] Li J. Ge L. Zhao Y. Zhai Y. Rao N. Yuan X. (2022a). TGF-β2 and TGF-β1 differentially regulate the odontogenic and osteogenic differentiation of mesenchymal stem cells. Arch. Oral Biol. 135, 105357. 10.1016/j.archoralbio.2022.105357 35085927

[B111] Li L. Liu P. Lv X. Yu T. Jin X. Wang R. (2022b). Ablation of FAM20C caused short root defects via suppressing the BMP signaling pathway in mice. J. Orofac. Orthop. 2022, 386. 10.1007/s00056-022-00386-7 35316352

[B112] Li Q. M. Li J. L. Feng Z. H. Lin H. C. Xu Q. (2020). Effect of histone demethylase KDM5A on the odontogenic differentiation of human dental pulp cells. Bioengineered 11, 449–462. 10.1080/21655979.2020.1743536 32208897 PMC7161540

[B113] Li Q. Yi B. Feng Z. Meng R. Tian C. Xu Q. (2018a). FAM20C could be targeted by TET1 to promote odontoblastic differentiation potential of human dental pulp cells. Cell Prolif. 51, e12426. 10.1111/cpr.12426 29277934 PMC6528884

[B114] Li R. Wang C. Tong J. Su Y. Lin Y. Zhou X. (2014). WNT6 promotes the migration and differentiation of human dental pulp cells partly through c-Jun N-terminal kinase signaling pathway. J. Endod. 40, 943–948. 10.1016/j.joen.2013.12.023 24935540

[B115] Li S. Kong H. Yao N. Yu Q. Wang P. Lin Y. (2011a). The role of runt-related transcription factor 2 (Runx2) in the late stage of odontoblast differentiation and dentin formation. Biochem. Biophys. Res. Commun. 410, 698–704. 10.1016/j.bbrc.2011.06.065 21703228

[B116] Li X. Yang G. Fan M. (2012). Effects of homeobox gene distal-less 3 on proliferation and odontoblastic differentiation of human dental pulp cells. J. Endod. 38, 1504–1510. 10.1016/j.joen.2012.07.009 23063225

[B117] Li X. Y. Ban G. F. Al-Shameri B. He X. Liang D. Z. Chen W. X. (2018b). High-temperature requirement protein A1 regulates odontoblastic differentiation of dental pulp cells via the transforming growth factor beta 1/smad signaling pathway. J. Endod. 44, 765–772. 10.1016/j.joen.2018.02.003 29580722

[B118] Li Y. Han D. Zhang H. Liu H. Wong S. Zhao N. (2015). Morphological analyses and a novel de novo DLX3 mutation associated with tricho-dento-osseous syndrome in a Chinese family. Eur. J. Oral Sci. 123, 228–234. 10.1111/eos.12197 26104267

[B119] Li Y. Lü X. Sun X. Bai S. Li S. Shi J. (2011b). Odontoblast-like cell differentiation and dentin formation induced with TGF-β1. Arch. Oral Biol. 56, 1221–1229. 10.1016/j.archoralbio.2011.05.002 21641578

[B120] Li Z. Yan M. Yu Y. Wang Y. Lei G. Pan Y. (2019). LncRNA H19 promotes the committed differentiation of stem cells from apical papilla via miR-141/SPAG9 pathway. Cell Death Dis. 10, 130. 10.1038/s41419-019-1337-3 30755596 PMC6372621

[B121] Lian M. Zhang Y. Shen Q. Xing J. Lu X. Huang D. (2016). JAB1 accelerates odontogenic differentiation of dental pulp stem cells. J. Mol. Histol. 47, 317–324. 10.1007/s10735-016-9672-5 26989054

[B122] Lim W. H. Liu B. Cheng D. Hunter D. J. Zhong Z. Ramos D. M. (2014). Wnt signaling regulates pulp volume and dentin thickness. J. Bone Min. Res. 29, 892–901. 10.1002/jbmr.2088 PMC4541795 23996396

[B123] Lin C. Zhang Q. Yu S. Lin Y. Li S. Liu H. (2018). miR-3065-5p regulates mouse odontoblastic differentiation partially through bone morphogenetic protein receptor type II. Biochem. Biophys. Res. Commun. 495, 493–498. 10.1016/j.bbrc.2017.11.026 29127007

[B124] Lin H. Liu H. Sun Q. Yuan G. Zhang L. Chen Z. (2013). KLF4 promoted odontoblastic differentiation of mouse dental papilla cells via regulation of DMP1. J. Cell Physiol. 228, 2076–2085. 10.1002/jcp.24377 23558921

[B125] Lin H. Xu L. Liu H. Sun Q. Chen Z. Yuan G. (2011a). KLF4 promotes the odontoblastic differentiation of human dental pulp cells. J. Endod. 37, 948–954. 10.1016/j.joen.2011.03.030 21689550

[B126] Lin M. Li L. Liu C. Liu H. He F. Yan F. (2011b). Wnt5a regulates growth, patterning, and odontoblast differentiation of developing mouse tooth. Dev. Dyn. 240, 432–440. 10.1002/dvdy.22550 21246660 PMC3023990

[B127] Lin Y. Xiao Y. Lin C. Zhang Q. Zhang S. Pei F. (2021). SALL1 regulates commitment of odontoblast lineages by interacting with RUNX2 to remodel open chromatin regions. Stem Cells 39, 196–209. 10.1002/stem.3298 33159702

[B128] Liu C. Yu J. Liu B. Liu M. Song G. Zhu L. (2022a). BACH1 regulates the proliferation and odontoblastic differentiation of human dental pulp stem cells. BMC Oral Health 22, 536. 10.1186/s12903-022-02588-2 36424585 PMC9694919

[B129] Liu C. Zhou N. Wang Y. Zhang H. Jani P. Wang X. (2018a). Abrogation of Fam20c altered cell behaviors and BMP signaling of immortalized dental mesenchymal cells. Exp. Cell Res. 363, 188–195. 10.1016/j.yexcr.2018.01.004 29337188 PMC5866767

[B130] Liu H. Lin H. Zhang L. Sun Q. Yuan G. Zhang L. (2013). miR-145 and miR-143 regulate odontoblast differentiation through targeting Klf4 and Osx genes in a feedback loop. J. Biol. Chem. 288, 9261–9271. 10.1074/jbc.M112.433730 23430263 PMC3610997

[B131] Liu M. Goldman G. Macdougall M. Chen S. (2022b). BMP signaling pathway in dentin development and diseases. Cells 11, 2216. 10.3390/cells11142216 35883659 PMC9317121

[B132] Liu Z. Chen T. Han Q. Chen M. You J. Fang F. (2018b). HDAC inhibitor LMK-235 promotes the odontoblast differentiation of dental pulp cells. Mol. Med. Rep. 17, 1445–1452. 10.3892/mmr.2017.8055 29138868 PMC5780081

[B133] Lopez-Cazaux S. Bluteau G. Magne D. Lieubeau B. Guicheux J. Alliot-Licht B. (2006). Culture medium modulates the behaviour of human dental pulp-derived cells: technical note. Eur. Cell Mater 11, 35–42. discussion 42. 10.22203/ecm.v011a05 16485235

[B134] Løvschall H. Tummers M. Thesleff I. Füchtbauer E. M. Poulsen K. (2005). Activation of the Notch signaling pathway in response to pulp capping of rat molars. Eur. J. Oral Sci. 113, 312–317. 10.1111/j.1600-0722.2005.00221.x 16048523

[B135] Lu X. Chen X. Xing J. Lian M. Huang D. Lu Y. (2019). miR-140-5p regulates the odontoblastic differentiation of dental pulp stem cells via the Wnt1/β-catenin signaling pathway. Stem Cell Res. Ther. 10, 226. 10.1186/s13287-019-1344-4 31358066 PMC6664499

[B136] Ma S. Liu G. Jin L. Pang X. Wang Y. Wang Z. (2016). IGF-1/IGF-1R/hsa-let-7c axis regulates the committed differentiation of stem cells from apical papilla. Sci. Rep. 6, 36922. 10.1038/srep36922 27833148 PMC5105129

[B137] Malik Z. Alexiou M. Hallgrimsson B. Economides A. N. Luder H. U. Graf D. (2018). Bone morphogenetic protein 2 coordinates early tooth mineralization. J. Dent. Res. 97, 835–843. 10.1177/0022034518758044 29489425

[B138] Marrelli M. Codispoti B. Shelton R. M. Scheven B. A. Cooper P. R. Tatullo M. (2018). Dental pulp stem cell mechanoresponsiveness: effects of mechanical stimuli on dental pulp stem cell behavior. Front. Physiol. 9, 1685. 10.3389/fphys.2018.01685 30534086 PMC6275199

[B139] Martens W. Bronckaers A. Politis C. Jacobs R. Lambrichts I. (2013). Dental stem cells and their promising role in neural regeneration: an update. Clin. Oral Investig. 17, 1969–1983. 10.1007/s00784-013-1030-3 23846214

[B140] Matsubara T. Suardita K. Ishii M. Sugiyama M. Igarashi A. Oda R. (2005). Alveolar bone marrow as a cell source for regenerative medicine: differences between alveolar and iliac bone marrow stromal cells. J. Bone Min. Res. 20, 399–409. 10.1359/JBMR.041117 15746984

[B141] Matsuoka K. Matsuzaka K. Yoshinari M. Inoue T. (2013). Tenascin-C promotes differentiation of rat dental pulp cells *in vitro* . Int. Endod. J. 46, 30–39. 10.1111/j.1365-2591.2012.02089.x 22747576

[B142] Mitchell F. N. Mitchell J. E. (1957). Vitamin-D-resistant rickets. AMA J. Dis. Child. 93, 385–390. 10.1001/archpedi.1957.02060040387005 13410374

[B143] Mitsiadis T. A. Feki A. Papaccio G. Catón J. (2011). Dental pulp stem cells, niches, and notch signaling in tooth injury. Adv. Dent. Res. 23, 275–279. 10.1177/0022034511405386 21677078

[B144] Miura M. Gronthos S. Zhao M. Lu B. Fisher L. W. Robey P. G. (2003). SHED: stem cells from human exfoliated deciduous teeth. Proc. Natl. Acad. Sci. U. S. A. 100, 5807–5812. 10.1073/pnas.0937635100 12716973 PMC156282

[B145] Morimoto M. Kérourédan O. Gendronneau M. Shuen C. Baradaran-Heravi A. Asakura Y. (2012). Dental abnormalities in Schimke immuno-osseous dysplasia. J. Dent. Res. 91, 29S–37s. 10.1177/0022034512450299 22699664 PMC3383106

[B146] Morsczeck C. Götz W. Schierholz J. Zeilhofer F. Kühn U. Möhl C. (2005). Isolation of precursor cells (PCs) from human dental follicle of wisdom teeth. Matrix Biol. 24, 155–165. 10.1016/j.matbio.2004.12.004 15890265

[B147] Mu C. Lv T. Wang Z. Ma S. Ma J. Liu J. (2014). Mechanical stress stimulates the osteo/odontoblastic differentiation of human stem cells from apical papilla via erk 1/2 and JNK MAPK pathways. Biomed. Res. Int. 2014, 494378. 10.1155/2014/494378 24826377 PMC4009119

[B148] Nieminen P. Lukinmaa P. L. Alapulli H. Methuen M. Suojärvi T. Kivirikko S. (2011). DLX3 homeodomain mutations cause tricho-dento-osseous syndrome with novel phenotypes. Cells Tissues Organs 194, 49–59. 10.1159/000322561 21252474

[B149] Nusse R. Clevers H. (2017). Wnt/β-Catenin signaling, disease, and emerging therapeutic modalities. Cell 169, 985–999. 10.1016/j.cell.2017.05.016 28575679

[B150] Nuti N. Corallo C. Chan B. M. Ferrari M. Gerami-Naini B. (2016). Multipotent differentiation of human dental pulp stem cells: a literature review. Stem Cell Rev. Rep. 12, 511–523. 10.1007/s12015-016-9661-9 27240827

[B151] Okawa R. Hamada M. Takagi M. Matayoshi S. Nakano K. (2022). A case of X-linked hypophosphatemic rickets with dentin dysplasia in mandibular third molars. Child. (Basel) 9, 1304. 10.3390/children9091304 PMC9497892 36138613

[B152] Olsson A. Matsson L. Blomquist H. K. Larsson A. Sjödin B. (1996). Hypophosphatasia affecting the permanent dentition. J. Oral Pathol. Med. 25, 343–347. 10.1111/j.1600-0714.1996.tb00274.x 8887081

[B153] Opsahl Vital S. Gaucher C. Bardet C. Rowe P. S. George A. Linglart A. (2012). Tooth dentin defects reflect genetic disorders affecting bone mineralization. Bone 50, 989–997. 10.1016/j.bone.2012.01.010 22296718 PMC3345892

[B154] Orikasa S. Kawashima N. Tazawa K. Hashimoto K. Sunada-Nara K. Noda S. (2022). Hypoxia-inducible factor 1α induces osteo/odontoblast differentiation of human dental pulp stem cells via Wnt/β-catenin transcriptional cofactor BCL9. Sci. Rep. 12, 682. 10.1038/s41598-021-04453-8 35027586 PMC8758693

[B155] Paino F. Ricci G. De Rosa A. D'Aquino R. Laino L. Pirozzi G. (2010). Ecto-mesenchymal stem cells from dental pulp are committed to differentiate into active melanocytes. Eur. Cell Mater 20, 295–305. 10.22203/ecm.v020a24 20931491

[B156] Palma-Lara I. Pérez-Ramírez M. García Alonso-Themann P. Espinosa-García A. M. Godinez-Aguilar R. Bonilla-Delgado J. (2021). FAM20C overview: classic and novel targets, pathogenic variants and raine syndrome phenotypes. Int. J. Mol. Sci. 22, 8039. 10.3390/ijms22158039 34360805 PMC8348777

[B157] Peng L. Ren L. B. Dong G. Wang C. L. Xu P. Ye L. (2010). Wnt5a promotes differentiation of human dental papilla cells. Int. Endod. J. 43, 404–412. 10.1111/j.1365-2591.2010.01693.x 20518933

[B158] Peng L. Ye L. Dong G. Ren L. B. Wang C. L. Xu P. (2009). WNT5A inhibits human dental papilla cell proliferation and migration. Biochem. Biophys. Res. Commun. 390, 1072–1078. 10.1016/j.bbrc.2009.10.136 19878652

[B159] Price J. A. Bowden D. W. Wright J. T. Pettenati M. J. Hart T. C. (1998a). Identification of a mutation in DLX3 associated with tricho-dento-osseous (TDO) syndrome. Hum. Mol. Genet. 7, 563–569. 10.1093/hmg/7.3.563 9467018

[B160] Price J. A. Wright J. T. Kula K. Bowden D. W. Hart T. C. (1998b). A common DLX3 gene mutation is responsible for tricho-dento-osseous syndrome in Virginia and North Carolina families. J. Med. Genet. 35, 825–828. 10.1136/jmg.35.10.825 9783705 PMC1051457

[B161] Qi S. Yan Y. Wen Y. Li J. Wang J. Chen F. (2017). The effect of delta-like 1 homologue on the proliferation and odontoblastic differentiation in human dental pulp stem cells. Cell Prolif. 50, e12335. 10.1111/cpr.12335 28205268 PMC6529117

[B162] Qin W. Lin Z. M. Deng R. Li D. D. Song Z. Tian Y. G. (2012a). p38a MAPK is involved in BMP-2-induced odontoblastic differentiation of human dental pulp cells. Int. Endod. J. 45, 224–233. 10.1111/j.1365-2591.2011.01965.x 21992459

[B163] Qin W. Liu P. Zhang R. Huang S. Gao X. Song Z. (2014). JNK MAPK is involved in BMP-2-induced odontoblastic differentiation of human dental pulp cells. Connect. Tissue Res. 55, 217–224. 10.3109/03008207.2014.882331 24409810

[B164] Qin W. Yang F. Deng R. Li D. Song Z. Tian Y. (2012b). Smad 1/5 is involved in bone morphogenetic protein-2-induced odontoblastic differentiation in human dental pulp cells. J. Endod. 38, 66–71. 10.1016/j.joen.2011.09.025 22152623

[B165] Raine J. Winter R. M. Davey A. Tucker S. M. (1989). Unknown syndrome: microcephaly, hypoplastic nose, exophthalmos, gum hyperplasia, cleft palate, low set ears, and osteosclerosis. J. Med. Genet. 26, 786–788. 10.1136/jmg.26.12.786 2614802 PMC1015765

[B166] Reibel A. Manière M. C. Clauss F. Droz D. Alembik Y. Mornet E. (2009). Orodental phenotype and genotype findings in all subtypes of hypophosphatasia. Orphanet J. Rare Dis. 4, 6. 10.1186/1750-1172-4-6 19232125 PMC2654544

[B167] Rodríguez-Lozano F. J. Bueno C. Insausti C. L. Meseguer L. Ramírez M. C. Blanquer M. (2011). Mesenchymal stem cells derived from dental tissues. Int. Endod. J. 44, 800–806. 10.1111/j.1365-2591.2011.01877.x 21477154

[B168] Sakai V. T. Zhang Z. Dong Z. Neiva K. G. Machado M. A. Shi S. (2010). SHED differentiate into functional odontoblasts and endothelium. J. Dent. Res. 89, 791–796. 10.1177/0022034510368647 20395410

[B169] Salmon B. Bardet C. Coyac B. R. Baroukh B. Naji J. Rowe P. S. (2014). Abnormal osteopontin and matrix extracellular phosphoglycoprotein localization, and odontoblast differentiation, in X-linked hypophosphatemic teeth. Connect. Tissue Res. 55 (1), 79–82. 10.3109/03008207.2014.923864 25158186

[B170] Salmon B. Bardet C. Khaddam M. Naji J. Coyac B. R. Baroukh B. (2013). MEPE-derived ASARM peptide inhibits odontogenic differentiation of dental pulp stem cells and impairs mineralization in tooth models of X-linked hypophosphatemia. PLoS One 8, e56749. 10.1371/journal.pone.0056749 23451077 PMC3579870

[B171] Schimke R. N. Horton W. A. King C. R. (1971). Chondroitin-6-sulphaturia, defective cellular immunity, and nephrotic syndrome. Lancet 2, 1088–1089. 10.1016/s0140-6736(71)90400-4 4106927

[B172] Seo B. M. Miura M. Gronthos S. Bartold P. M. Batouli S. Brahim J. (2004). Investigation of multipotent postnatal stem cells from human periodontal ligament. Lancet 364, 149–155. 10.1016/S0140-6736(04)16627-0 15246727

[B173] Shang W. Xiong S. (2022). Phenytoin is promoting the differentiation of dental pulp stem cells into the direction of odontogenesis/osteogenesis by activating BMP4/smad pathway. Dis. Markers 2022, 7286645. 10.1155/2022/7286645 35493301 PMC9050280

[B174] Sheng R. Wang Y. Wu Y. Wang J. Zhang S. Li Q. (2021). METTL3-Mediated m(6) A mRNA methylation modulates tooth root formation by affecting NFIC translation. J. Bone Min. Res. 36, 412–423. 10.1002/jbmr.4180 32936965

[B175] Shi R. Yang H. Lin X. Cao Y. Zhang C. Fan Z. (2019). Analysis of the characteristics and expression profiles of coding and noncoding RNAs of human dental pulp stem cells in hypoxic conditions. Stem Cell Res. Ther. 10, 89. 10.1186/s13287-019-1192-2 30867055 PMC6417198

[B176] Simon S. Resch H. Klaushofer K. Roschger P. Zwerina J. Kocijan R. (2018). Hypophosphatasia: from diagnosis to treatment. Curr. Rheumatol. Rep. 20, 69. 10.1007/s11926-018-0778-5 30203264

[B177] Simpson M. A. Hsu R. Keir L. S. Hao J. Sivapalan G. Ernst L. M. (2007). Mutations in FAM20C are associated with lethal osteosclerotic bone dysplasia (Raine syndrome), highlighting a crucial molecule in bone development. Am. J. Hum. Genet. 81, 906–912. 10.1086/522240 17924334 PMC2265657

[B178] Simpson M. A. Scheuerle A. Hurst J. Patton M. A. Stewart H. Crosby A. H. (2009). Mutations in FAM20C also identified in non-lethal osteosclerotic bone dysplasia. Clin. Genet. 75, 271–276. 10.1111/j.1399-0004.2008.01118.x 19250384

[B179] Smith Z. D. Meissner A. (2013). DNA methylation: roles in mammalian development. Nat. Rev. Genet. 14, 204–220. 10.1038/nrg3354 23400093

[B180] Song Y. Liu X. Feng X. Gu Z. Gu Y. Lian M. (2017). NRP1 accelerates odontoblast differentiation of dental pulp stem cells through classical wnt/β-catenin signaling. Cell Reprogr. 19, 324–330. 10.1089/cell.2017.0020 28910136

[B181] Song Y. Wang C. Gu Z. Cao P. Huang D. Feng G. (2019). CKIP-1 suppresses odontoblastic differentiation of dental pulp stem cells via BMP2 pathway and can interact with NRP1. Connect. Tissue Res. 60, 155–164. 10.1080/03008207.2018.1483355 29852799

[B182] Sonoyama W. Liu Y. Fang D. Yamaza T. Seo B. M. Zhang C. (2006). Mesenchymal stem cell-mediated functional tooth regeneration in swine. PLoS One 1, e79. 10.1371/journal.pone.0000079 17183711 PMC1762318

[B183] Souza M. A. Soares Junior L. A. Santos M. A. Vaisbich M. H. (2010). Dental abnormalities and oral health in patients with Hypophosphatemic rickets. Clin. (Sao Paulo) 65, 1023–1026. 10.1590/s1807-59322010001000017 PMC2972601 21120305

[B184] Spranger J. Hinkel G. K. Stöss H. Thoenes W. Wargowski D. Zepp F. (1991). Schimke immuno-osseous dysplasia: a newly recognized multisystem disease. J. Pediatr. 119, 64–72. 10.1016/s0022-3476(05)81040-6 2066860

[B185] Steinhart Z. Angers S. (2018). Wnt signaling in development and tissue homeostasis. Development 145, dev146589. 10.1242/dev.146589 29884654

[B186] Su T. Zhu Y. Wang X. Zhu Q. Duan X. (2023). Hereditary dentin defects with systemic diseases. Oral Dis. 29, 2376–2393. 10.1111/odi.14589 37094075

[B187] Sui B. Wu D. Xiang L. Fu Y. Kou X. Shi S. (2020). Dental pulp stem cells: from discovery to clinical application. J. Endod. 46, S46–s55. 10.1016/j.joen.2020.06.027 32950195

[B188] Sun D. G. Xin B. C. Wu D. Zhou L. Wu H. B. Gong W. (2017). miR-140-5p-mediated regulation of the proliferation and differentiation of human dental pulp stem cells occurs through the lipopolysaccharide/toll-like receptor 4 signaling pathway. Eur. J. Oral Sci. 125, 419–425. 10.1111/eos.12384 29130547

[B189] Sun Q. Liu H. Chen Z. (2015). The fine tuning role of microRNA-RNA interaction in odontoblast differentiation and disease. Oral Dis. 21, 142–148. 10.1111/odi.12237 24654877

[B190] Sun Q. Liu H. Lin H. Yuan G. Zhang L. Chen Z. (2013). MicroRNA-338-3p promotes differentiation of mDPC6T into odontoblast-like cells by targeting Runx2. Mol. Cell Biochem. 377, 143–149. 10.1007/s11010-013-1580-3 23380982

[B191] Sun Z. Yu S. Chen S. Liu H. Chen Z. (2019). SP1 regulates KLF4 via SP1 binding motif governed by DNA methylation during odontoblastic differentiation of human dental pulp cells. J. Cell Biochem. 120, 14688–14699. 10.1002/jcb.28730 31009133 PMC8895433

[B192] Tai T. F. Chan C. P. Lin C. C. Chen L. I. Jeng J. H. Chang M. C. (2008). Transforming growth factor beta2 regulates growth and differentiation of pulp cells via ALK5/Smad2/3. J. Endod. 34, 427–432. 10.1016/j.joen.2008.02.007 18358889

[B193] Tamura M. Nemoto E. (2016). Role of the Wnt signaling molecules in the tooth. Jpn. Dent. Sci. Rev. 52, 75–83. 10.1016/j.jdsr.2016.04.001 28408959 PMC5390339

[B194] Tang J. Saito T. (2018). Elucidation on predominant pathways involved in the differentiation and mineralization of odontoblast-like cells by selective blockade of mitogen-activated protein kinases. Biomed. Res. Int. 2018, 2370438. 10.1155/2018/2370438 29675422 PMC5838463

[B195] Tao H. Li Q. Lin Y. Zuo H. Cui Y. Chen S. (2020). Coordinated expression of p300 and HDAC3 upregulates histone acetylation during dentinogenesis. J. Cell Biochem. 121, 2478–2488. 10.1002/jcb.29470 31692090 PMC7808212

[B196] Tao H. Lin H. Sun Z. Pei F. Zhang J. Chen S. (2019). Klf4 promotes dentinogenesis and odontoblastic differentiation via modulation of TGF-β signaling pathway and interaction with histone acetylation. J. Bone Min. Res. 34, 1502–1516. 10.1002/jbmr.3716 PMC8895434 31112333

[B197] Taskiran E. Z. Karaosmanoglu B. Koşukcu C. Doğan Ö A. Taylan-Şekeroğlu H. Şimşek-Kiper P. (2017). Homozygous indel mutation in CDH11 as the probable cause of Elsahy-Waters syndrome. Am. J. Med. Genet. A 173, 3143–3152. 10.1002/ajmg.a.38495 28988429

[B198] Thesleff I. (2003). Epithelial-mesenchymal signalling regulating tooth morphogenesis. J. Cell Sci. 116, 1647–1648. 10.1242/jcs.00410 12665545

[B199] Tomazelli K. B. Modolo F. Trentin A. G. Garcez R. C. Biz M. T. (2015). Temporo-spatial analysis of Osterix, HNK1 and Sox10 during odontogenesis and maxillaries osteogenesis. Tissue Cell 47, 465–470. 10.1016/j.tice.2015.07.007 26253417

[B200] Tu S. Wu J. Chen L. Tian Y. Qin W. Huang S. (2020). LncRNA CALB2 sponges miR-30b-3p to promote odontoblast differentiation of human dental pulp stem cells via up-regulating RUNX2. Cell Signal 73, 109695. 10.1016/j.cellsig.2020.109695 32565162

[B201] Vijaykumar A. Root S. H. Mina M. (2021). Wnt/β-Catenin signaling promotes the formation of preodontoblasts *in vitro* . J. Dent. Res. 100, 387–396. 10.1177/0022034520967353 33103548 PMC7989141

[B202] Wang B. Li H. Liu Y. Lin X. Lin Y. Wang Y. (2014a). Expression patterns of WNT/β-CATENIN signaling molecules during human tooth development. J. Mol. Histol. 45, 487–496. 10.1007/s10735-014-9572-5 24647585

[B203] Wang B. L. Wang Z. Nan X. Zhang Q. C. Liu W. (2019). Downregulation of microRNA-143-5p is required for the promotion of odontoblasts differentiation of human dental pulp stem cells through the activation of the mitogen-activated protein kinases 14-dependent p38 mitogen-activated protein kinases signaling pathway. J. Cell Physiol. 234, 4840–4850. 10.1002/jcp.27282 30362514

[B204] Wang C. Ren L. Peng L. Xu P. Dong G. Ye L. (2010). Effect of Wnt6 on human dental papilla cells *in vitro* . J. Endod. 36, 238–243. 10.1016/j.joen.2009.09.007 20113781

[B205] Wang C. Song Y. Gu Z. Lian M. Huang D. Lu X. (2018). Wedelolactone enhances odontoblast differentiation by promoting wnt/β-catenin signaling pathway and suppressing NF-κB signaling pathway. Cell Reprogr. 20, 236–244. 10.1089/cell.2018.0004 30089027

[B206] Wang D. Zhu N. Xie F. Qin M. Wang Y. (2022). Long noncoding RNA IGFBP7-AS1 promotes odontogenesis of stem cells from human exfoliated deciduous teeth via the p38 MAPK pathway. Stem Cells Int. 2022, 9227248. 10.1155/2022/9227248 35469296 PMC9034958

[B207] Wang H. Lindborg C. Lounev V. Kim J. H. Mccarrick-Walmsley R. Xu M. (2016). Cellular hypoxia promotes heterotopic ossification by amplifying BMP signaling. J. Bone Min. Res. 31, 1652–1665. 10.1002/jbmr.2848 PMC5010462 27027798

[B208] Wang T. Liu H. Ning Y. Xu Q. (2014b). The histone acetyltransferase p300 regulates the expression of pluripotency factors and odontogenic differentiation of human dental pulp cells. PLoS One 9, e102117. 10.1371/journal.pone.0102117 25007265 PMC4090168

[B209] Wang X. He F. Tan Y. Tian W. Qiu S. (2011). Inhibition of Delta1 promotes differentiation of odontoblasts and inhibits proliferation of human dental pulp stem cell *in vitro* . Arch. Oral Biol. 56, 837–845. 10.1016/j.archoralbio.2011.02.006 21392732

[B210] Wang X. Wang S. Lu Y. Gibson M. P. Liu Y. Yuan B. (2012). FAM20C plays an essential role in the formation of murine teeth. J. Biol. Chem. 287, 35934–35942. 10.1074/jbc.M112.386862 22936805 PMC3476261

[B211] Wei M. Zhang C. Tian Y. Du X. Wang Q. Zhao H. (2020). Expression and function of WNT6: from development to disease. Front. Cell Dev. Biol. 8, 558155. 10.3389/fcell.2020.558155 33425886 PMC7794017

[B212] Whyte M. P. (2016). Hypophosphatasia - aetiology, nosology, pathogenesis, diagnosis and treatment. Nat. Rev. Endocrinol. 12, 233–246. 10.1038/nrendo.2016.14 26893260

[B213] Whyte M. P. Zhang F. Wenkert D. Mcalister W. H. Mack K. E. Benigno M. C. (2015). Hypophosphatasia: validation and expansion of the clinical nosology for children from 25 years experience with 173 pediatric patients. Bone 75, 229–239. 10.1016/j.bone.2015.02.022 25731960

[B214] Winters R. W. Graham J. B. Williams T. F. Mc F. V. Burnett C. H. (1958). A genetic study of familial hypophosphatemia and vitamin D resistant rickets with a review of the literature. Med. Baltim. 37, 97–142. 10.1097/00005792-195805000-00001 13565132

[B215] Woo S. M. Seong K. J. Oh S. J. Park H. J. Kim S. H. Kim W. J. (2015). 17β-Estradiol induces odontoblastic differentiation via activation of the c-Src/MAPK pathway in human dental pulp cells. Biochem. Cell Biol. 93, 587–595. 10.1139/bcb-2015-0036 26393498

[B216] Wright J. T. Kula K. Hall K. Simmons J. H. Hart T. C. (1997). Analysis of the tricho-dento-osseous syndrome genotype and phenotype. Am. J. Med. Genet. 72, 197–204. 10.1002/(sici)1096-8628(19971017)72:2<197::aid-ajmg14>3.3.co;2-0 9382143

[B217] Wright J. T. Roberts M. W. Wilson A. R. Kudhail R. (1994). Tricho-dento-osseous syndrome. Features of the hair and teeth. Oral Surg. Oral Med. Oral Pathol. 77, 487–493. 10.1016/0030-4220(94)90228-3 8028872

[B218] Wu A. Bao Y. Yu H. Zhou Y. Lu Q. (2019). Berberine accelerates odontoblast differentiation by wnt/β-catenin activation. Cell Reprogr. 21, 108–114. 10.1089/cell.2018.0060 30969881

[B219] Wu H. Yang L. Chen L. L. (2017). The diversity of long noncoding RNAs and their generation. Trends Genet. 33, 540–552. 10.1016/j.tig.2017.05.004 28629949

[B220] Xiao G. Jiang D. Gopalakrishnan R. Franceschi R. T. (2002). Fibroblast growth factor 2 induction of the osteocalcin gene requires MAPK activity and phosphorylation of the osteoblast transcription factor, Cbfa1/Runx2. J. Biol. Chem. 277, 36181–36187. 10.1074/jbc.M206057200 12110689

[B221] Xiao Y. Lin Y. X. Cui Y. Zhang Q. Pei F. Zuo H. Y. (2021). Zeb1 promotes odontoblast differentiation in a stage-dependent manner. J. Dent. Res. 100, 648–657. 10.1177/0022034520982249 33419386

[B222] Xin T. Li Q. Bai R. Zhang T. Zhou Y. Zhang Y. (2021). A novel mutation of SATB2 inhibits odontogenesis of human dental pulp stem cells through Wnt/β-catenin signaling pathway. Stem Cell Res. Ther. 12, 595. 10.1186/s13287-021-02660-8 34863303 PMC8642962

[B223] Xu C. Xie X. Zhao L. Wu Y. Wang J. (2022). The critical role of nuclear factor I-C in tooth development. Oral Dis. 28, 2093–2099. 10.1111/odi.14046 34637578

[B224] Xu J. Yu B. Hong C. Wang C. Y. (2013). KDM6B epigenetically regulates odontogenic differentiation of dental mesenchymal stem cells. Int. J. Oral Sci. 5, 200–205. 10.1038/ijos.2013.77 24158144 PMC3967319

[B225] Xu K. Xiao J. Zheng K. Feng X. Zhang J. Song D. (2018). MiR-21/STAT3 signal is involved in odontoblast differentiation of human dental pulp stem cells mediated by TNF-α. Cell Reprogr. 20, 107–116. 10.1089/cell.2017.0042 29620442

[B226] Yamashiro T. Zheng L. Shitaku Y. Saito M. Tsubakimoto T. Takada K. (2007). Wnt10a regulates dentin sialophosphoprotein mRNA expression and possibly links odontoblast differentiation and tooth morphogenesis. Differentiation 75, 452–462. 10.1111/j.1432-0436.2006.00150.x 17286598

[B227] Yamaza T. Kentaro A. Chen C. Liu Y. Shi Y. Gronthos S. (2010). Immunomodulatory properties of stem cells from human exfoliated deciduous teeth. Stem Cell Res. Ther. 1, 5. 10.1186/scrt5 20504286 PMC2873699

[B228] Yang G. Li X. Yuan G. Liu P. Fan M. (2014). The effects of osterix on the proliferation and odontoblastic differentiation of human dental papilla cells. J. Endod. 40, 1771–1777. 10.1016/j.joen.2014.04.012 25258338

[B229] Yang J. Ye L. Hui T. Q. Yang D. M. Huang D. M. Zhou X. D. (2015). Bone morphogenetic protein 2-induced human dental pulp cell differentiation involves p38 mitogen-activated protein kinase-activated canonical WNT pathway. Int. J. Oral Sci. 7, 95–102. 10.1038/ijos.2015.7 26047580 PMC4817555

[B230] Yang S. Liu Q. Chen S. Zhang F. Li Y. Fan W. (2022). Extracellular vesicles delivering nuclear factor I/C for hard tissue engineering: treatment of apical periodontitis and dentin regeneration. J. Tissue Eng. 13, 20417314221084095. 10.1177/20417314221084095 35321254 PMC8935403

[B231] Yang Y. Zhao Y. Liu X. Chen Y. Liu P. Zhao L. (2017). Effect of SOX2 on odontoblast differentiation of dental pulp stem cells. Mol. Med. Rep. 16, 9659–9663. 10.3892/mmr.2017.7812 29039570

[B232] Yokose S. Naka T. (2010). Lymphocyte enhancer-binding factor 1: an essential factor in odontoblastic differentiation of dental pulp cells enzymatically isolated from rat incisors. J. Bone Min. Metab. 28, 650–658. 10.1007/s00774-010-0185-0 20425127

[B233] Yu M. Jiang Z. Wang Y. XI Y. Yang G. (2021). Molecular mechanisms for short root anomaly. Oral Dis. 27, 142–150. 10.1111/odi.13266 31883171

[B234] Yu S. Li J. Zhao Y. Li X. Ge L. (2020). Comparative secretome analysis of mesenchymal stem cells from dental apical papilla and bone marrow during early odonto/osteogenic differentiation: potential role of transforming growth factor-β2. Front. Physiol. 11, 41. 10.3389/fphys.2020.00041 32210829 PMC7073820

[B235] Yuan H. Suzuki S. Terui H. Hirata-Tsuchiya S. Nemoto E. Yamasaki K. (2022). Loss of IκBζ drives dentin formation via altered H3K4me3 status. J. Dent. Res. 101, 951–961. 10.1177/00220345221075968 35193410

[B236] Yue J. Wu B. Gao J. Huang X. Li C. Ma D. (2012). DMP1 is a target of let-7 in dental pulp cells. Int. J. Mol. Med. 30, 295–301. 10.3892/ijmm.2012.982 22552299

[B237] Yun H. M. Chang S. W. Park K. R. Herr L. Kim E. C. (2016). Combined effects of growth hormone and mineral trioxide aggregate on growth, differentiation, and angiogenesis in human dental pulp cells. J. Endod. 42, 269–275. 10.1016/j.joen.2015.08.020 26435469

[B238] Yusufzai T. Kadonaga J. T. (2008). HARP is an ATP-driven annealing helicase. Science 322, 748–750. 10.1126/science.1161233 18974355 PMC2587503

[B239] Zeng L. Sun S. Dong L. Liu Y. Liu H. Han D. (2019). DLX3 epigenetically regulates odontoblastic differentiation of hDPCs through H19/miR-675 axis. Arch. Oral Biol. 102, 155–163. 10.1016/j.archoralbio.2019.04.009 31029881

[B240] Zeng L. Sun S. Han D. Liu Y. Liu H. Feng H. (2018a). Long non-coding RNA H19/SAHH axis epigenetically regulates odontogenic differentiation of human dental pulp stem cells. Cell Signal 52, 65–73. 10.1016/j.cellsig.2018.08.015 30165103

[B241] Zeng L. Zhao N. Han D. Liu H. Liu Y. Wang Y. (2017). DLX3 mutation negatively regulates odontogenic differentiation of human dental pulp cells. Arch. Oral Biol. 77, 12–17. 10.1016/j.archoralbio.2017.01.011 28135572

[B242] Zeng L. Zhao N. Li F. Han D. Liu Y. Liu H. (2018b). miR-675 promotes odontogenic differentiation of human dental pulp cells by epigenetic regulation of DLX3. Exp. Cell Res. 367, 104–111. 10.1016/j.yexcr.2018.03.035 29604248

[B243] Zhai Y. Wang Y. Rao N. Li J. Li X. Fang T. (2019). Activation and biological properties of human β defensin 4 in stem cells derived from human exfoliated deciduous teeth. Front. Physiol. 10, 1304. 10.3389/fphys.2019.01304 31695620 PMC6817489

[B244] Zhai Y. Yuan X. Zhao Y. Ge L. Wang Y. (2020). Potential application of human β-defensin 4 in dental pulp repair. Front. Physiol. 11, 1077. 10.3389/fphys.2020.01077 32973567 PMC7472722

[B245] Zhang C. Chang J. Sonoyama W. Shi S. Wang C. Y. (2008). Inhibition of human dental pulp stem cell differentiation by Notch signaling. J. Dent. Res. 87, 250–255. 10.1177/154405910808700312 18296609

[B246] Zhang H. Wang J. Deng F. Huang E. Yan Z. Wang Z. (2015a). Canonical Wnt signaling acts synergistically on BMP9-induced osteo/odontoblastic differentiation of stem cells of dental apical papilla (SCAPs). Biomaterials 39, 145–154. 10.1016/j.biomaterials.2014.11.007 25468367 PMC4258144

[B247] Zhang J. Wang Z. Jiang Y. Niu Z. Fu L. Luo Z. (2015b). Nuclear Factor I-C promotes proliferation and differentiation of apical papilla-derived human stem cells *in vitro* . Exp. Cell Res. 332, 259–266. 10.1016/j.yexcr.2015.01.020 25668322

[B248] Zhang J. Wu J. Lin X. Liu X. (2022). Platelet-rich fibrin promotes the proliferation and osteo-/odontoblastic differentiation of human dental pulp stem cells. Curr. Stem Cell Res. Ther. 18, 560–567. 10.2174/1574888X17666220704092411 35794740

[B249] Zhang L. Zhao J. Dong J. Liu Y. Xuan K. Liu W. (2021a). GSK3β rephosphorylation rescues ALPL deficiency-induced impairment of odontoblastic differentiation of DPSCs. Stem Cell Res. Ther. 12, 225. 10.1186/s13287-021-02235-7 33823913 PMC8022410

[B250] Zhang Q. Shi S. Liu Y. Uyanne J. Shi Y. Shi S. (2009). Mesenchymal stem cells derived from human gingiva are capable of immunomodulatory functions and ameliorate inflammation-related tissue destruction in experimental colitis. J. Immunol. 183, 7787–7798. 10.4049/jimmunol.0902318 19923445 PMC2881945

[B251] Zhang R. Lin J. Liu Y. Yang S. He Q. Zhu L. (2021b). Transforming growth factor-β signaling regulates tooth root dentinogenesis by cooperation with wnt signaling. Front. Cell Dev. Biol. 9, 687099. 10.3389/fcell.2021.687099 34277628 PMC8277599

[B252] Zhang S. Li X. Wang S. Yang Y. Guo W. Chen G. (2020a). Immortalized Hertwig's epithelial root sheath cell line works as model for epithelial-mesenchymal interaction during tooth root formation. J. Cell Physiol. 235, 2698–2709. 10.1002/jcp.29174 31512758

[B253] Zhang S. Yang Y. Jia S. Chen H. Duan Y. Li X. (2020b). Exosome-like vesicles derived from Hertwig's epithelial root sheath cells promote the regeneration of dentin-pulp tissue. Theranostics 10, 5914–5931. 10.7150/thno.43156 32483427 PMC7254987

[B254] Zhang W. Walboomers X. F. Shi S. Fan M. Jansen J. A. (2006). Multilineage differentiation potential of stem cells derived from human dental pulp after cryopreservation. Tissue Eng. 12, 2813–2823. 10.1089/ten.2006.12.2813 17518650

[B255] Zhang X. Ning T. Wang H. Xu S. Yu H. Luo X. (2019). Stathmin regulates the proliferation and odontoblastic/osteogenic differentiation of human dental pulp stem cells through Wnt/β-catenin signaling pathway. J. Proteomics 202, 103364. 10.1016/j.jprot.2019.04.014 31009804

[B256] Zhang Y. D. Chen Z. Song Y. Q. Liu C. Chen Y. P. (2005). Making a tooth: growth factors, transcription factors, and stem cells. Cell Res. 15, 301–316. 10.1038/sj.cr.7290299 15916718

[B257] Zhao N. Zeng L. Liu Y. Han D. Liu H. Xu J. (2017). DLX3 promotes bone marrow mesenchymal stem cell proliferation through H19/miR-675 axis. Clin. Sci. (Lond) 131, 2721–2735. 10.1042/CS20171231 28963438

[B258] Zhong J. Tu X. Kong Y. Guo L. Li B. Zhong W. (2020). LncRNA H19 promotes odontoblastic differentiation of human dental pulp stem cells by regulating miR-140-5p and BMP-2/FGF9. Stem Cell Res. Ther. 11, 202. 10.1186/s13287-020-01698-4 32460893 PMC7251819

[B259] Zhou M. Kawashima N. Suzuk N. Yamamoto M. Ohnishi K. Katsube K. (2015). Periostin is a negative regulator of mineralization in the dental pulp tissue. Odontology 103, 152–159. 10.1007/s10266-014-0152-7 24647621

[B260] Zhu N. Wang D. Xie F. Qin M. Wang Y. (2022). MiR-335-3p/miR-155-5p involved in IGFBP7-AS1-enhanced odontogenic differentiation. Int. Dent. J. 73, 362–369. 10.1016/j.identj.2022.07.008 35999071 PMC10213769

